# Analysis of Diffusion Coefficients of Iron Monoboride and Diiron Boride Coating Formed on the Surface of AISI 420 Steel by Two Different Models: Experiments and Modelling

**DOI:** 10.3390/ma16134801

**Published:** 2023-07-03

**Authors:** Martín Ortiz-Domínguez, Ángel Jesús Morales-Robles, Oscar Armando Gómez-Vargas, Teresita de Jesús Cruz-Victoria

**Affiliations:** 1 Ingeniería Mecánica, Escuela Superior de Ciudad Sahagún, Universidad Autónoma del Estado de Hidalgo, Carretera Cd. Sahagún–Otumba s/n, Zona Industrial, Ciudad Sahagun 43990, Hidalgo, Mexico; 2 Área Académica de Ciencias de la Tierra y Materiales, Instituto de Ciencias Básicas e Ingeniería, Universidad Autónoma del Estado de Hidalgo, Carretera Pachuca–Tulancingo Km. 4.5 s/n, Col. Carboneras, Mineral de la Reforma 42184, Hidalgo, Mexico; 3 División de Estudios de Posgrado e Investigación, Instituto Tecnológico de Tlalnepantla, TecNM, Avenida Instituto Tecnológico s/n. Col. La Comunidad, Tlalnepantla de Baz 54070, Estado de Mexico, Mexico

**Keywords:** powder-pack boronizing, propagation of the boride coatings, diffusion models, hardness, activation energy of FeB and Fe_2_B, wear resistance

## Abstract

In the present work, two mathematical diffusion models have been used to estimate the growth of the iron monoboride and diiron boride coating formed on AISI 420 steel. The boronizing of the steel was carried out with the solid diffusion packing method at a boronizing temperature of 1123 K–1273 K. Experimental results show the two-coating system consists of an outer monoboride and an inner diiron boride coating with a predominantly planar structure at the propagation front. The depth of the boride coating increases according to temperature and treatment time. A parabolic curve characterizes the propagation of the boride coatings. The two proposed mathematical models of mass transfer diffusion are founded on the solution corresponding to Fick’s second fundamental law. The first is based on a linear boron concentration–penetration profile without time dependence, and the second model with time dependence (exact solution). For both models, the theoretical law of parabolic propagation and the average flux of boron atoms (Fick’s first fundamental law) at the growth interfaces (monoboride/diiron boride and diiron boride/substrate) are considered to estimate the propagation of the boride coatings (monoboride and diiron boride). To validate the mathematical models, a programming code is written in the MATLAB program (adaptation 7.5) designed to simulate the growth of the boride coatings (monoboride and diiron boride). The following parameters are used as input data for this computer code: (the layer thicknesses of the FeB and Fe_2_B phases, the operating temperature, the boronizing time, initial formation time of the boride coating, the surface boron concentration limits, FeB/Fe_2_B and Fe_2_B/Fe growth interfaces, and the mass transfer diffusion coefficient of boron in the iron monoboride and diiron boride phases). The outputs of the computer code are the constants $\varepsilon_{FeB}$ and $\varepsilon_{Fe_{2}B}$. The assessment of activation energies of AISI 420 steel for the two mathematical models of mass transfer is coincident ($Q_{FeB}=$221.9 kJ∙mol^−1^ and $Q_{Fe_{2}B}=$209.1 kJ∙mol^−1^). A numerical analysis was performed using a standard Taylor series for clarification of the proximity between the two models. SEM micrographs exhibited a strong propensity toward a flat-fronted composition at expansion interfaces of the iron monoboride and diiron boride coating, confirmed by XRD analysis. Tribological characterizations included the Vickers hardness test method, pin-on-disc, and Daimler–Benz Rockwell-C indentation adhesion tests. After thorough analysis, the energies were compared to the existing literature to validate our experiment. We found that our models and experimental results agreed. The diffusion models we utilized were crucial in gaining a deeper understanding of the boronizing behavior of AISI 420 steel, and they also allowed us to predict the thicknesses of the iron monoboride and diiron boride coating. These models provide helpful approaches for predicting the behavior of these steels.

## 1. Introduction

The thermochemical surface-hardening treatment known as boronizing is used to improve metal components’ service life and performance. The process is used to increase resistance to corrosion and abrasive wear, significantly increase surface hardness, and decrease the coefficient of friction. Time and temperature are essential in boronizing [1,2,3,4,5,6]. Powder-pack boronizing is an economical process for hardening steels used in the metal-mechanical industry. It is a simple, low-cost process, and handling many components is unnecessary. This process generally uses a boron-rich mixture [3]. Also, the chemical constitution of the steel influences the microstructure and the attributes of the boronized coatings [2,4]. Thus, the material to be hardened is immersed in the container with the powder, then heated in a muffle-type furnace subject to a temperature within the range of 1123 K–1273 K and a period. After the chemical reaction of the mixture, the boron atoms are released and they diffuse from the surface into the substrate. This results in the formation of one or two layers of iron borides [4]. 

Most steels, except those with high aluminum or silicon content, can undergo the boronizing process [3,4]. This includes low-carbon steels, such as AISI 1010, AISI 1011, AISI 1018, AISI 1020, and ASTM A–36, which contain between 0.08 and 0.25 wt.% carbon. These steels are known for being soft but malleable, meaning they can be shaped into wire and strands without breaking. Additionally, they are very workable, making them easy to deform, cut, machine, and weld. They are used in the automotive industry, pipelines, structural elements of buildings and bridges, reinforcing rods, ship hulls, etc. Medium-carbon steels, between 0.25–0.60% by weight of carbon (AISI 1045, AISI 4140), are more robust than low-carbon steel but less ductile (the ability to be shaped into wires and threads without breaking). They are used in the manufacture of parts requiring high mechanical and wear resistance (gears, shafts, bearing applications). High-carbon steel is mainly used to manufacture tools due to its strength and durability. High-carbon steel typically contains between 0.60–1.40% carbon by weight, making it even more potent than medium-carbon steel. However, it is also less malleable, meaning it is less able to be shaped into wire and strands without breaking. To increase its hardness, other elements are often added to form carbides, such as tungsten alloy steels, which contain in their chemical composition a certain proportion of various elements to improve unique physical, mechanical, or chemical properties (AISI 4340, AISI 8620, and AISI 9840). The elements added may be carbon, chromium, molybdenum, vanadium, copper, tungsten, cobalt, columbium, or nickel, in quantities exceeding the minimum amounts established, in addition to more significant portions of manganese and silicon than in carbon steels such as stainless steels; it is the addition of chromium that gives these steels their stainless characteristic (AISI 303, AISI 304, AISI 310, AISI 316, AISI 410, AISI 416, AISI 420, and AISI 430). In oxidizing media such as air, chromium forms a very thin and compact oxide layer that insulates the material from corrosive attacks. 

In the case of ARMCO^®^ iron [7,8,9,10], low-alloy, and all-carbon steels [11,12,13], the growth fronts (diiron boride/substrate interface) tend to show a needle-like microstructure, a columnar of dendritic nature, as was reported by Ninham and Hutchings [14]. The columnarity of the diiron boride/substrate interface is a product of the growth of adjacent arms, much like the solidification of alloys (ferrous and non-ferrous) [15]. Likewise, Palombarini and Carbucicchio [16] in the same way reported that the sawtooth shape at the growth interface (diiron boride/substrate) in ARMCO^®^ iron and low-alloy steels is due to enhanced growing at the peaks of the iron boride needles. On the other hand, Martini and Palombarini [17] proposed a study of the growing mechanism of the iron monoboride and diiron boride phases. From observations of metallographic images and X-ray diffraction profile, they concluded that the diiron boride phase is the first to form considering three stages of development (stage 1: growth of circular crystals with random orientations; stage 2: interaction between crystals, which are forced to grow towards the interior of the substrate; stage 3: establishment of a texture with a defined crystallographic orientation (002)). The effect of the alloying elements of the substrate in combination with the boron atoms directly influences the tribological properties. A prolonged time and elevated temperature in the boronizing process implies an increase in the thickness of the boride coating. In a two-layer system (iron monoboride + diiron boride), the iron monoboride phase with a boron (B) content of 16.4% has a high hardness compared to the Fe_2_B phase with 9% boron content [10]. Tsipas et al. [18] reported that carbon is insoluble in the FeB phase and has a significantly low solubility in the diiron boride phase. 

A carbon atom is displaced to the growth interface (Fe_2_B/substrate) and, in combination with boron, forms borocementite (Fe_3_(B_0.67_C_0.33_)) [19]. This is the main cause of the circular morphology of iron borides and the decreasing of the thickness of the boride coating [20,21]. For kinetic modeling of the boronizing treatment, numerous approaches have been proposed for iron-based alloys treated to obtain the optimization of their coating thicknesses to satisfy industrial requirements. For example, Campos et al. [22] determined the boron diffusion coefficients in the diiron boride phase and the diffusion zone for AISI 1045 steel using a paste-boronizing process. Likewise, they selected the diffusion coefficients of boron in iron monoboride, diiron boride phases, and the diffusion zone in AISI M2 steel, considering a mathematical linear boron concentration profile, the mass balance equation at the interfaces, and the theoretical law of parabolic propagation. Yu et al. [23] investigated the growth kinetics of iron monoboride and diiron boride boronized coatings formed on an AISI 1018 mild steel with 0.2% of carbon content, using a simple diffusion model to depict the event, considering the reduction in boron concentration during the powder-pack boronizing treatment by Spark Plasma Sintering. Torres et al. [24] proposed a dimensional mathematical model related to the Buckingham-Pi theorem to study the power of the growth laws of organized coatings formed on the surface of AISI 1045 and M2 steels. The dimensional model (Buckingham-Pi theorem) provides a method for computing sets of dimensionless parameters involved in the paste-boronizing treatment. The model was validated with experimental data (average thickness of the monoboride, and diiron boride coatings, the thickness of the boron paste on the substrate surface, treatment time, growing constant and boron (a chemical element with the symbol B) concentrations at the surface and interface).

Dybkov [25] conducted a comprehensive study on forming boride coatings on different substrates using two differential equations. Samples of other steels were embedded in a boron-rich mixture containing 5% potassium tetrafluoroborate as an activator (KBF_4_). Six steels with varying percentages of chromium (5%, 10%, 13%, 15%, 25% and 30%) were considered in the study; the boronizing temperature range was from 1123 K to 1223 K with extended treatment times of up to 12 h.

Another interesting study is the one proposed by Doñu et al. [26]; in their work, they used a simple diffusion model to evaluate the diffusion coefficients of boron in iron monoboride and diiron boride coatings formed on the surface of a tungsten-molybdenum alloyed high-speed steel, particularly satisfactory for cutting tools where edge maintenance and high toughness with good wear resistance are required (AISI M2 steel), considering a linear concentration profile for both phases formed. They assumed that the layers obey the theoretical law of parabolic growth and established two mass conservation equations at the growth interfaces. The boronizing method used was powder-pack with dry boron paste. 

Campos-Silva et al. [27] studied the formation of iron monoboride, diiron boride, and diffusion zone (DZ) coatings from the solution of Fick’s second law without time dependence (a linear profile of boron concentration through the three phases) and three mass conservation equations at the iron monoboride/diiron boride, diiron boride/DZ, and DZ/substrate and incorporating the effects of iron boride incubation time, in an austenitic, antimagnetic, non-quenchable, non-temperable stainless steel with good ductility and weldability properties (AISI 316L).

Nait Abdellah and Keddam [28] formulated two mass conservation equations at the iron monoboride/diiron boride and diiron boride/substrate growth interfaces to study the dynamics of monoboride and diiron boride coating on AISI M2 steel. In the proposed mathematical mass transfer model, they assumed a nonlinear boron concentration profile from the solution of Fick’s second law through the boride coatings and consideration of the incubation times of iron borides. Keddam and Kulka [29] proposed an innovation to study the dynamics of monoboride and diiron boride coatings formed in a high wear-resistant tool steel used in cutting, stamping, stamping, forming rolls, porcelain molds and refractories (AISI D2). The mathematical diffusion model is based on the Goodman method, from the solution obtained after solving the Differential-Algebraic System of Equations, which validated the experimental data. Zong et al. [30] proposed a simple mass transfer mathematical model to analyze the formation of boride layers developed on the surface of martensitic stainless steel used for the fabrication of bearings (X65Cr14) by a solid diffusion boriding process for different treatment times and temperatures. They also examined the main mechanical properties of the boride coatings obtained. Finally, it is essential to address the influence and consequences of grain boundaries on mass transfer phenomena. It has become of the utmost importance to the design of materials used in the metalworking industry due to their direct impact on various mechanical properties, highlighting hardness and corrosion resistance; for example, Azeem et al. [31] created six Symmetrically Tilted Grain Boundary models (STGBs) to determine the influence of the Grain Boundary (GB) on the phosphorus (P) atom transfer phenomenon.

In this study, we used two mathematical diffusion models to compare the diffusion coefficients obtained for the boride coatings of iron monoboride and diiron boride formed created on the surface of stainless steel (AISI 420 steel). The first model is a linear model that does not depend on time, while the second model is an exact solution that considers time dependence. After analyzing the outcomes of these two models, we can gain a deeper insight into the growth kinetics of boride layers and determine which model is more effective. AISI 420 steel is martensitic stainless steel that can be quenched and tempered. It has an excellent response and, therefore, can reach hardness values between 494–567 HV, providing good compression resistance. It is used to manufacture molds (plastic injection, compression, blowing, extrusion, and glass) and cutlery for making knives, gears, valve components, pumps, and shafts for the food industry. In plastic injection molds, excellent polishing properties, wear, and corrosion resistance are required even at high oxidation temperatures (approximately 773 K). Due to its chemical composition (see Section 3), it is used in processes where the injected material is very corrosive and abrasive, for example, injection of chlorinated polymers, such as polyvinyl chloride, acetates, and some types of bakelites. AISI 420 steel can also be hardened through the thermochemical treatment of nitriding, but it has yet to give good results when the component is subjected to corrosive media; for that reason, the thermochemical boronizing treatment prolongs the service life of the mechanical element under extreme operating conditions. It improves its anti-wear properties and corrosion resistance in acidic and alkaline media by forming a compact surface-hardened layer [2,3,4]. This is why more studies are needed on the boronizing of stainless steel (AISI 420 steel). 

In the first model, from the solution of Fick’s second law a linear boron concentration profile was considered through the boride coatings for both phases; the growth of the coatings obeyed a parabolic growth for both phases, and the average flux mass of boron (B) atoms (Fick’s first law) at the propagation interfaces (iron monoboride/diiron boride and diiron boride/substrate) is shown. In the second approach, the error function with time dependence (the exact solution of Fick’s second law) was assumed as the boron concentration profile along both phases. For this purpose, the solid diffusion packing method in the boronizing process was applied for the boronizing process in stainless steel (AISI 420 steel) subject to a temperature range from 1123 K to 1223 K. The influence of boronizing temperature and time on the boride coatings was examined. This study is a consulting tool to understand the boronizing behavior of AISI 420 steel in depth. Likewise, it offers an innovative approach to calculating the coating thicknesses of boride coatings. Furthermore, the bilayer’s metallurgical and tribological surface characteristics (FeB + Fe_2_B) were investigated.

## 2. Boronizing Properties of Iron and Steel

**Figure 1:**
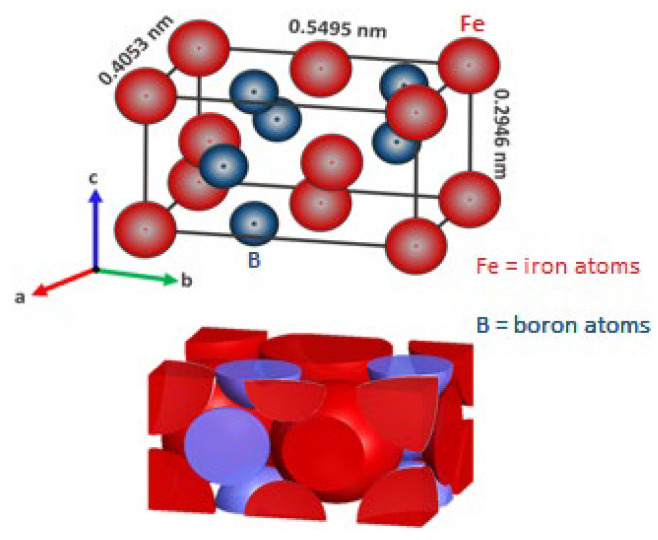
The orthorhombic unit cell of the FeB phase with axial lengths: (a = 405.3 × 10^−12^ m, b = 549.5 × 10^−12^ m, c = 294.6 × 10^−12^ m).

The effect of the chemical elements that make up the substrate in combination with the boron atoms tend to form a sawtooth morphology or a flat growth front and are usually composed of:

(i) An outer FeB layer which has an orthorhombic crystal lattice containing ~16.4% boron that is composed of 8 atoms in a cell (with four iron atoms and four boron atoms) with axial lengths: a = 405.3 × 10^−12^ m, b = 549.5 × 10^−12^ m, c = 294.6 × 10^−12^ m (see Figure 1).
(ii) With a microhardness range of approximately 1900–2100 Vickers Hardness, the melting point of iron monoboride is about 1663 K, Young’s modulus is about 590 × 10^3^ MPa, the density is $\rho_{FeB}=$ 6.7 × 10^3^ kg∙m^–3^, its molecular mass $M_{FeB}=$ 66.6 g∙mol^−1^, and the coefficient of thermal expansion is 84 × 10^–9^ K^−1^ in the temperature range of about 473–873 K. An important quality to note is that the FeB phase is chemically stable up to 1723 K in the Fe–B system [6].

**Figure 2:**
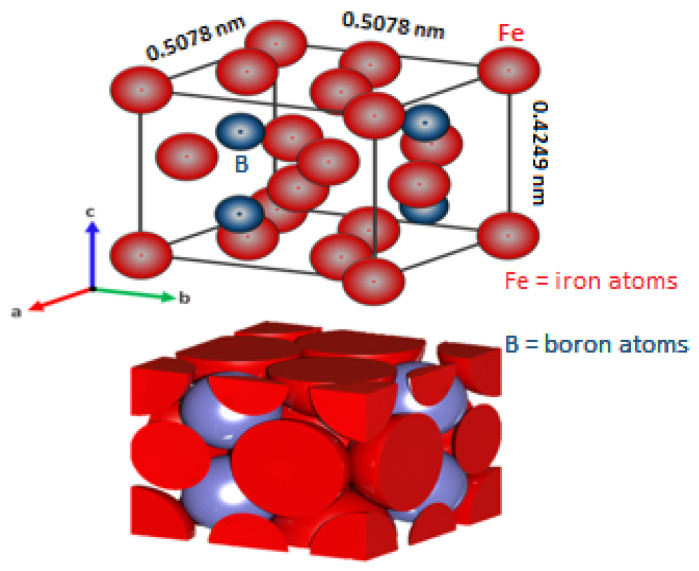
The tetragonal unit cell of the Fe_2_B phase with axial lengths: a = 507.8 × 10^−12^ m, b = 507.8 × 10^−12^ m, c = 424.9 × 10^−12^ m.

An inner Fe_2_B layer has a tetragonal crystal lattice containing ~ 9% boron that is composed of twelve atoms in the crystalline cell with these axial lengths: a = 507.8 × 10^−12^ m, c = 424.9 × 10^−12^ m, c/a = 831 × 10^−12^ m (see Figure 2). With a microhardness of about 1800–2000 HV, Young’s modulus is about (285–295) × 10^3^ MPa, the density is $\rho_{Fe_{2}B}=$7.3 × 10^3^ kg∙m^–3^, its molecular mass $M_{Fe_{2}B}=$ 122.5 g∙mol^−1^, and the coefficient of thermal expansion is 29 × 10^–9^ K^−1^ in the temperature range of about 473–873 K [6]. The crystal structures depicted in Figure 1 (single orthorhombic body-centered of the FeB phase) and Figure 2 (tetragonal body-centered of the Fe_2_B phase) were modeled by using VESTA (Visualization for Electronic and Structural Analysis) software [32]. The initial positions of the iron and boron atoms were looked up in the tables of the Open Crystallography Database [33] with folio numbers 1,010,477 for the iron monoboride phase and 1,511,152 for the diiron boride phase, respectively.

**Figure 3:**
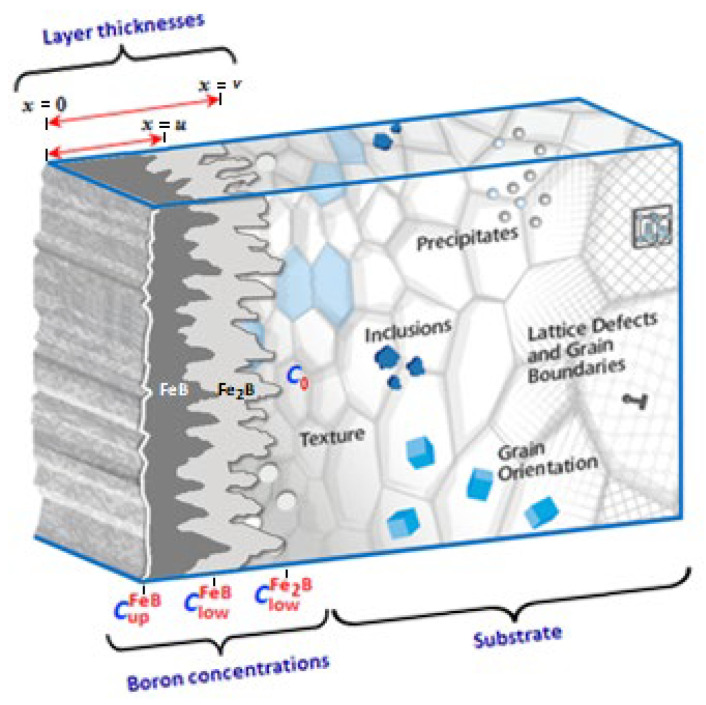
Schematic of the boride coatings (FeB + Fe_2_B) located on the surface of the ferrous alloy (AISI 420 steel).

A typical microstructure of a two-layer system (FeB + Fe_2_B) is shown in Figure 3. As mentioned above, the iron monoboride phase is slightly harder and more compact than the diiron boride phase; it is considered more brittle and prone to crack formation. The boride coatings develop on the surface of the ferrous alloy through a nucleation period (incubation time) [34,35], i.e., the initial coating propagation is limited to specific sites on the substrate surface that grow and merge to form a very thin initial iron boride layer. At this stage, the initial layer is randomly oriented [17]. The layer growth is strongly favored along the preferential direction [002] for FeB and Fe_2_B [17,35,36,37]. The boron concentration at the FeB phase surface is denoted by $C_{up}^{FeB}$ (=16.4 wt. %B) [27,29,38,39,40,41], the boron concentration at the FeB/Fe_2_B growth interface is $C_{low}^{FeB}$ (=16.2 wt. %B) [27,29,37,38,39,40,41], the concentration at the diiron boride/ferrous or non-ferrous alloy interface is $C_{low}^{Fe_{2}B}$ (=8.8 wt. %B) [27,29,34,38,39,40,41], and $C_{0}$ (=3.5 × 10^–5^ wt. %B) symbolizes the concentration value of boron solubility in the ferrous and non-ferrous alloy and could be neglected [42,43].

## 3. Resources and Techniques

### 3.1. Resources and Experimental Method

**Figure 4:**
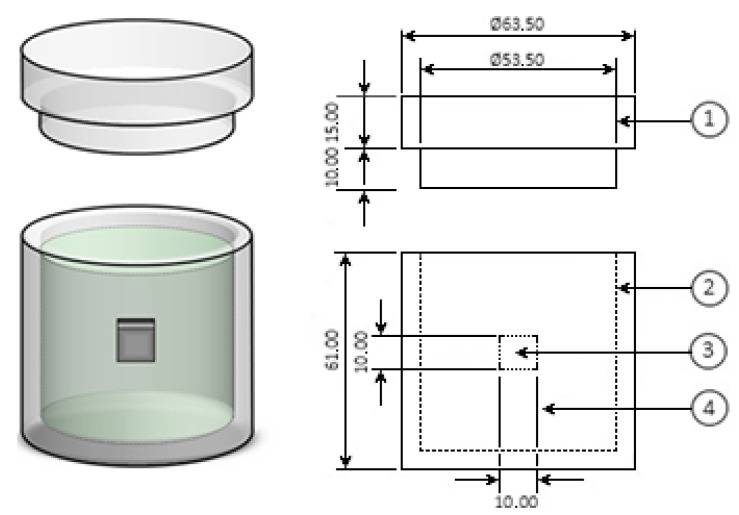
Schematic representation of the cylindrical container made of AISI 316L steel for powder boronizing treatment (1: lid; 2: boron-rich powder mixture (boron carbide + potassium tetrafluoroborate + silicon carbide); 3: steel sample; 4: container) (millimeter scale).

A stainless-steel alloy (AISI 420) was used as the base material for applying the boronizing thermochemical treatment. The chemical components of the substrate are as follows: 0.35% Carbon, 0.54% Manganese, 0.56% Silicon, 13.71% Chromium, 0.09% Vanadium, 0.08% Molybdenum, 0.02% Tungsten, 0.02% Phosphorus, 0.01% Sulfur, 0.3% Nickel, 0.002% Aluminum, 0.01% Cobalt, 0.02% Copper, 0.02% Niobium, 0.005% Titanium, 0.001% Lead, 0.01% Tin, 0.001% Zinc, 0.01% Nitrogen and 84.2% Iron [44]. The combination of its properties makes it suitable for applications using corrosive mold materials, e.g., injection molding of chlorinated polymers such as polyvinyl chloride and acetate. It is also suitable in hot runner molds, subject to intense atmospheric humidity and injection of abrasive polymers such as, for example, thermosetting plastics (bakelite) with filler and other reinforcement. It is also suitable for molds in the glass and optics industry. It can also be used in cutlery, surgical instrumentation, valve components and pumps, shafts, and other structural components. The samples were machined to 1 cm × 1 cm × 1 cm. Before applying the thermochemical process, all specimens were roughened with silicon carbide sandpaper with a grain size in the range of 80 to 2500; the samples were subjected to an ultrasonic bath to remove surface impurities (Bandelin SONOREX RK 52 H (Bruckstr, Burladingen, Germany)) with acetone or propanone (C_3_H_6_O) for a time of 3 min. Subsequently, the samples were packed with the boron-rich mixture of 335 × 10^−1^ wt.% boron carbide as a main source of boron (B_4_C), 54 × 10^−1^ wt.% potassium tetrafluoroborate as an activator (KBF_4_), and 611 × 10^−1^ wt.% silicon carbide (SiC) as a diluent in a cylindrical container made of medical grade AISI 316L steel (see Figure 4).

The samples were hardened in the 1123 K to 1223 K boronizing temperature range, and the treatment time was 2 h to 8 h using a conventional electric flask of the brand Nabertherm N 250/85 HA (Bahnhofstr, Lilienthal, Germany). Once the treatment temperature was selected within the mentioned range and the time was reached, the container was placed at room temperature.

### 3.2. Feature Tools

**Figure 5:**
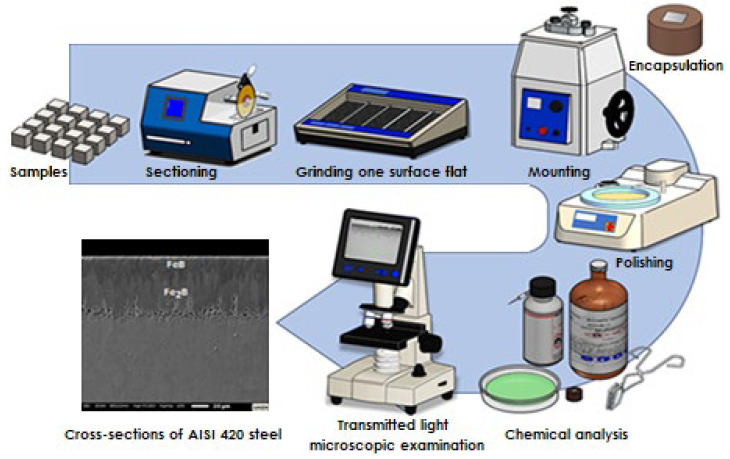
Schematic representation of the standard ASTM E3–11 metallographic sample preparation procedure [45].

After applying the boronizing treatment, the samples were sectioned, then roughened with silicon carbide paper to level and obtain a mirror-like surface. Subsequently, Buehler SimpliMet XPS1 mounting equipment (Hong Kong, SAR, China) was used to encapsulate the samples considering temperature, pressure, and heating and cooling time. The encapsulated samples were then polished with a magnetically supported polishing cloth (Buehler) using aluminum oxide abrasive (3 and 1 µm, Buehler), followed by polishing with diamond suspension (25 × 10^–7^ and 5 × 10^–8^ m, Buehler Ltd.) for 20 min with different sizes to remove surface deformation coatings and expose the material structure for analysis. Between the polishing steps, to eliminate external agents, the samples were subjected to an ultrasonic bath with acetone for three minutes. Nital was used as a solution to reveal the microstructure of the treated samples and consisted of 3% nitric acid and 97% methyl alcohol. The depth of the boronized coating was examined by a 4K optical microscope, VHX–7000 series (Higashi-Yodogawa-ku, Osaka, Japan). All the steps of the metallographic procedure can be seen in Figure 5. An automatic process was carried out using specialized Image Pro Plus 6.3 software to determine the depth of the boride coatings (iron monoboride + diiron boride). Sixteen samples were considered for the growth kinetics study, in addition to one replicate for each.

**Figure 6:**
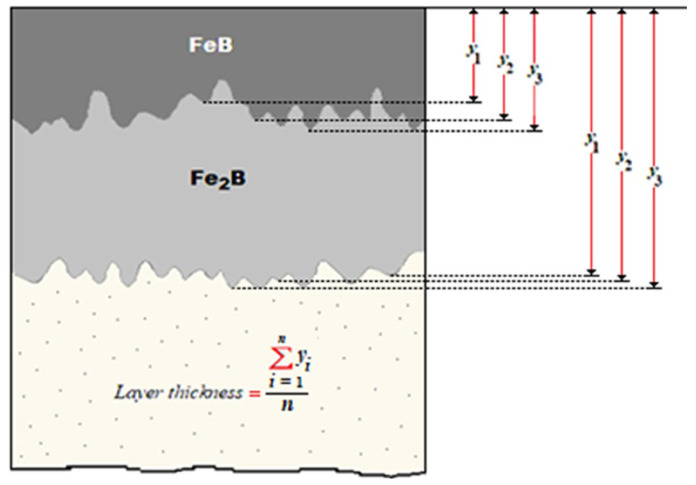
Illustrative drawing of the measurement of the average values of the FeB and Fe_2_B phase layer thicknesses.

Also, fifty measurements in different cross-sections were made to determine the mean values of the depths of the iron monoboride and diiron boride coatings (refer to Figure 6).

Verification of the iron monoboride and diiron boride iron boride phases was through the grazing angle X-ray diffraction technique. The different peaks in the XRD diffraction data were analyzed using MATCh crystallographic software, employing a diffraction radiation type CoKα with a wavelength λ = 0.18 nm and a scanning range of 25 to 85 with an Inel Equinox 2000 Diffractometer (Waltham, MA, USA). The microhardness profile was measured through the FeB and Fe_2_B layers with a Vickers DuraScan 20 G5 micro-durometer (Kellau, Kuchl, Austria) under a load of 4.903 × 10^−1^ Newtons for 15 s. Verification of the adhesion performance of the boronized coatings was through the Daimler–Benz Rockwell-C proofing; for this the VDI3198 standard was followed [46], and the maximum load of 1475 Newtons was applied, which allowed for observing the deterioration around the crater formed by the indentation [47]. Three indentations were performed on each boronized specimen to evaluate the quality of the boride coatings formed on the surface of the substrate by adhesive bonding. The craters scars formed due to the indentation were scanned with a JEOL SEM (Scanning Electron Microscope) model JSM–7800 FEG with a resolution of 0.7 nm (Akishima, Tokyo, Japan). Regarding the tribological performance of the boronized coatings and the ferrous and non-ferrous alloy, a pin-on-disc analysis was carried out, which refers to sliding wear in dry conditions and without lubrication, considering a constant sliding distance of 8 × 10^2^ m, a constant linear velocity of 80 × 10^–3^ mm/s, a radial distance equal to 1.4 × 10^–2^ m, a test load equal to 5 Newtons, and a relative humidity of 45 volume% at room temperature. AISI 52,100 bearing steel balls, with high wear resistance and rolling fatigue resistance characteristics, were used to slide against the AISI 420 boronized steel surface. The balls used in this test had a constant radius of 2.375 mm, a microhardness of approximately of 850 HV, and an average roughness of Ra = 8 × 10^–9^ m. A CSM tribometer (Need–ham Heights, MA, USA) was used for the test. The penetration of the wear track generated on the boronized surface was estimated using a Mitutoyo Surftest SJ–301 diamond-tipped surface roughness tester (Takatsu-ku, Kawasaki, Japan).

### 3.3. First Mathematical Model: Linear Diffusion Model without Time Dependence (Steady-State)

**Figure 7:**
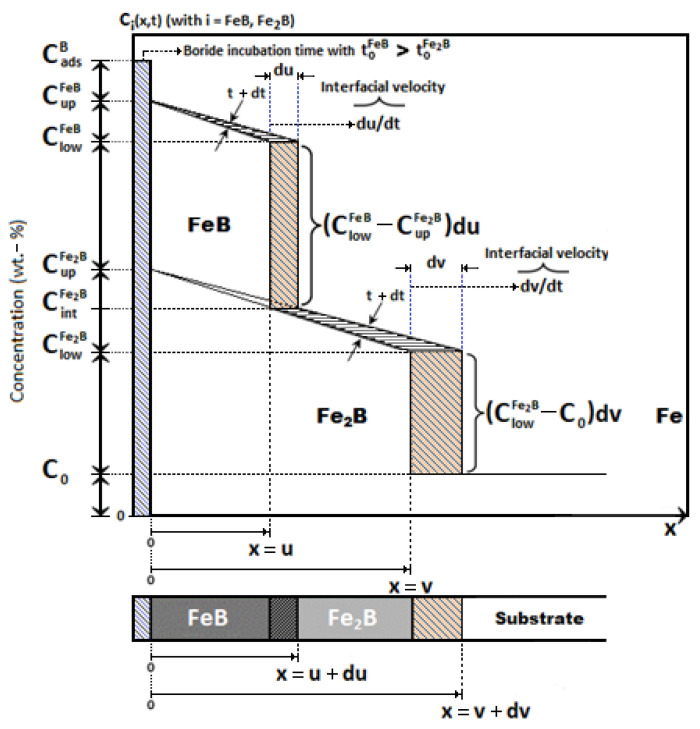
Graphical illustration of boron distribution profiles through the iron monoboride and diiron boride phases.

The linear diffusion mathematical model [22], without time dependence, was implemented to calculate the minimum activation energies and diffusion coefficients of the iron monoboride and diiron boride phases in AISI 420 stainless steel alloy. Figure 7 represents the boron distribution profiles along the iron monoboride and diiron boride coatings, respectively. *u* symbolizes the depth of the coating corresponding to the iron monoboride phase, v symbolizes the depth of the coating corresponding to the diiron boride phase,$C_{int}^{Fe_{2}B}$ illustrates the boron concentration at the iron monoboride/diiron boride interface [48,49], and is determined from the boron concentration profile $C_{Fe_{2}B}(x)$. The surface $C_{up}^{FeB}$ and $C_{low}^{FeB}$ interface concentration limits for the iron monoboride phase exhibit a very small concentration range (of approximately one atomic percent boron), and similarly, for the Fe_2_B phase limits ($C_{up}^{Fe_{2}B}$ and $C_{low}^{Fe_{2}B}$ as determined by Brakman et al. [19]). The accumulation of boron adsorbed ($C_{ads}^{B}$) on the substrate surface was determined by Yu et al. [23].

The mathematical model of mass transfer considers a series of assumptions which address its validity in conjunction with experimental data and theoretical reasoning [28,29,50]:

- After a particular incubation time, the first iron borides nucleate and form a primitive coating.
- The iron monoboride and diiron boride coatings grow as a result of perpendicular diffusion of boron atoms on the surface of the ferrous and non-ferrous alloys.
- Boron atoms obtained from the chemical reaction flow in one direction only.
- Boron concentrations at the growth interfaces (FeB/Fe_2_B, Fe_2_B/substrate) and surface remain unchanged during the process.
- The FeB and Fe_2_B boride layers form under conditions of thermodynamic equilibrium, i.e., constant temperature.
- The iron monoboride and diiron boride coatings follow the theoretical law of parabolic propagation.
- A planar morphology is assumed at the iron monoboride/diiron boride and diiron boride/substate growth interfaces.

In the mass transfer problem, the boundary conditions and the initial condition were established.

Assuming these conditions, we can express the initial and boundary conditions as follows (see Figure 3):

Initial condition (*t* = 0):

(1) $$C_{i}\left(x\right)=0,\;\mathrm{for}\;x>0\;{\;\mathrm{with}\;i=\mathrm{FeB},\;\mathrm{Fe}}_{2}B,$$

Boundary conditions:

(2) $$C_{FeB}\left(x=0\right)=C_{up}^{FeB},\;\mathrm{for}\;C_{ads}^{B}>16.40\;\mathrm{wt}.\%,$$

(3) $$C_{FeB}\left(x=u\right)=C_{low}^{FeB},\;\mathrm{for}\;C_{ads}^{B}<16.23\;\mathrm{wt}.\%,$$

(4) $$C_{Fe_{2}B}\left(x=l\right)=\;C_{int}^{Fe_{2}B},\;\mathrm{for}\;C_{ads}^{B}>9.0\;\mathrm{wt}.\%.$$

with l=v − u

(5) $$C_{Fe_{2}B}\left(x=v\right)=C_{low}^{Fe_{2}B},\;\mathrm{for}\;C_{ads}^{B}<8.83\;\mathrm{wt}.\%.$$

#### 3.3.1. Phase Fe_2_B

The solution of Fick’s second law without time dependence is a mathematical expression describing a substance’s diffusion in a stationary medium. It is based on the principle of mass conservation and considers the concentration gradient of the substance, i.e.:

(6) $$\nabla^{2}C_{Fe_{2}B}(x)=0,$$

and in Equations (4) and (5), the following mathematical expression determines the boron distribution profile along the diiron boride phase:

(7) $$C_{Fe_{2}B}(x)=C_{int}^{Fe_{2}B}+\frac{C_{low}^{Fe_{2}B}-C_{int}^{Fe_{2}B}}{v\;-\;u}\left(x-u\right).$$

On the other hand, the formulation of the mass conservation equation at the diiron boride/substate growth interface considers the incoming flow without time dependence as: ${\vec{J}}_{Fe_{2}B}{\left[x(t)\right]}_{x\;=\;v}\hat{i}=-D_{Fe_{2}B}{\left\{dC_{Fe_{2}B}\left[x(t)\right]/dx\right\}}_{x\;=\;v}\hat{i}$ and also to the outflow without time dependence as: ${\vec{J}}_{Fe}{\left[x(t+dt)\right]}_{x\;=\;v+dv}\hat{i}=-D_{Fe}{\left\{dC_{Fe}\left[x(t+dt)\right]/dx\right\}}_{x\;=\;v+dv}\hat{i}=0$. Thus the equation can be written as follows:

(8) $$\left(\frac{C_{low}^{Fe_{2}B}-2C_{0}+C_{int}^{Fe_{2}B}}{2}\right){\left(\frac{dx}{dt}\right)|}_{x\;=\;v}\hat{i}=-D_{Fe_{2}B}{\frac{dC_{Fe_{2}B}\left(x\right)}{dx}|}_{x\;=\;v}\hat{i}.$$

The layer thickness for the FeB phase is represented by Equation (9):

(9) $$u\left(t\right)=2\varepsilon_{FeB}D_{FeB}^{1/2}{\left(t-t_{0}^{FeB}\right)}^{1/2},$$

where $\varepsilon_{FeB}$ is a dimensionless constant, and $t_{0}^{FeB}$ corresponds to the incubation time of iron boride for the FeB phase. Likewise, the layer thickness for the Fe_2_B phase is represented by Equation (10):

(10) $$v\left(t\right)=2\varepsilon_{Fe_{2}B}D_{Fe_{2}B}^{1/2}{\left(t-t_{0}^{Fe_{2}B}\right)}^{1/2},$$

where $\varepsilon_{Fe_{2}B}$ is a dimensionless constant, and $t_{0}^{Fe_{2}B}$ corresponds to the incubation time of iron boride for the Fe_2_B phase. Substituting Equations (7) and (10) into Equation (8), the following is obtained:

(11) $$\left(\frac{C_{low}^{Fe_{2}B}-2C_{0}+C_{int}^{Fe_{2}B}}{2}\right)\frac{\varepsilon_{Fe_{2}B}D_{Fe_{2}B}^{1/2}}{{\left(t-t_{0}^{Fe_{2}B}\right)}^{1/2}}=-D_{Fe_{2}B}\left(\frac{C_{low}^{Fe_{2}B}-C_{int}^{Fe_{2}B}}{v-u}\right).$$

Figure 7 shows the following equivalence of the slopes in the Fe_2_B phase is observed.

(12) $$\frac{C_{low}^{Fe_{2}B}-C_{up}^{Fe_{2}B}}{v}=\frac{C_{int}^{Fe_{2}B}-C_{up}^{Fe_{2}B}}{u}=\frac{C_{low}^{Fe_{2}B}-C_{int}^{Fe_{2}B}}{v-u}.$$

Substituting Equation (12) in Equation (11) is obtained:

(13) $$\left(\frac{C_{low}^{Fe_{2}B}-2C_{0}+C_{int}^{Fe_{2}B}}{2}\right)\frac{\varepsilon_{Fe_{2}B}}{{\left(t-t_{0}^{Fe_{2}B}\right)}^{1/2}}=-D_{Fe_{2}B}^{1/2}\left(\frac{C_{low}^{Fe_{2}B}-C_{up}^{Fe_{2}B}}{v}\right).$$

Finally, we can determine $\varepsilon_{Fe_{2}B}$ as follows:

(14) $$\varepsilon_{Fe_{2}B}^{2}=\frac{C_{up}^{Fe_{2}B}-C_{low}^{Fe_{2}B}}{C_{low}^{Fe_{2}B}-2C_{0}+C_{int}^{Fe_{2}B}}.$$

#### 3.3.2. Phase FeB

For the case of the formulation of the mass conservation equation at the iron monoboride/diiron boride growth interface, consider the incoming flow without time dependence as: ${\vec{J}}_{FeB}{\left[x(t)\right]}_{x\;=\;u}\hat{i}=-D_{FeB}{\left\{dC_{FeB}\left[x(t)\right]/dx\right\}}_{x\;=\;u}\hat{i}$ and the outflow also without time dependence as: ${\vec{J}}_{FeB}{\left[x(t+dt)\right]}_{x\;=\;u+du}\hat{i}=-D_{Fe_{2}B}{\left\{dC_{Fe_{2}B}\left[x(t+dt)\right]/dx\right\}}_{x\;=\;u+du}\hat{i}.$ Thus the equation can be written as follows:

(15) $$\left(\frac{C_{up}^{FeB}-2C_{int}^{Fe_{2}B}+C_{low}^{FeB}}{2}\right){\left(\frac{dx}{dt}\right)|}_{x\;=\;u}\hat{i}=-D_{FeB}{\frac{dC_{FeB}\left(x\right)}{dx}|}_{x\;=\;u}\hat{i}-\left(-D_{Fe_{2}B}{\frac{dC_{Fe_{2}B}\left(x\right)}{dx}|}_{x\;=\;u+du}\right)\hat{i}$$

Similarly to the previous case, the fundamental solution of Fick’s second law without time dependence is written as follows:

(16) $$\nabla^{2}C_{FeB}=0,$$

and from Equations (3) and (4), the boron distribution profile along the iron monoboride phase is obtained, which has the following form:

(17) $$C_{FeB}(x)=\frac{C_{low}^{FeB}-C_{up}^{FeB}}{u}x+C_{up}^{FeB}.$$

Substituting Equations (7), (9), (12) and (17) into Equation (15), we obtain

(18) $$\left(\frac{C_{up}^{FeB}-2C_{int}^{Fe_{2}B}+C_{low}^{FeB}}{2}\right)\left(2\varepsilon_{FeB}D_{FeB}^{1/2}\right)\hat{i}=-D_{FeB}\left(\frac{C_{low}^{FeB}-C_{up}^{FeB}}{\varepsilon_{FeB}D_{FeB}^{1/2}}\right)\hat{i}+D_{Fe_{2}B}\left(\frac{C_{int}^{Fe_{2}B}-C_{up}^{Fe_{2}B}}{\varepsilon_{FeB}D_{FeB}^{1/2}}\right)\hat{i}.$$

We define the slopes from Equations (9) and (10):

(19) $$\kappa_{1}^{2}=4\varepsilon_{Fe_{2}B}^{2}D_{Fe_{2}B},$$

(20) $$\kappa_{2}^{2}=4\varepsilon_{FeB}^{2}D_{FeB},$$

Combining Equations (18)–(20), we obtain the following expression for $\varepsilon_{FeB}^{2}$:

(21) $$\varepsilon_{FeB}^{2}=\frac{\varepsilon_{Fe_{2}B}^{2}\left(C_{up}^{FeB}-C_{low}^{FeB}\right)\kappa_{2}^{2}}{\varepsilon_{Fe_{2}B}^{2}\left(C_{up}^{FeB}-2\;C_{int}^{Fe_{2}B}+C_{low}^{FeB}\right)\kappa_{2}^{2}-\kappa_{1}^{2}\left(C_{int}^{Fe_{2}B}-C_{up}^{Fe_{2}B}\right)\kappa_{2}^{2}}.$$

### 3.4. Second Mathematical Model: Diffusion Model with Time Dependence

The mathematical expression of the second Fick law to determine concentration profiles for the FeB and Fe_2_B phases is:

(22) $$\frac{\partial^{2}C_{Fe_{2}B}\left(x,t\right)}{\partial x^{2}}=\frac{1}{D_{Fe_{2}B}}\frac{\partial C_{Fe_{2}B}\left(x,t\right)}{\partial t}.$$

With solving Equation (22), the exact solution is obtained [51,52]:

(23) $$C_{i}\left(x,t\right)=A_{i}+B_{i}erf\left(\frac{x}{2\sqrt{D_{i}\left(t-t_{0}^{i}\right)}}\right),\;\mathrm{with}\;i=FeB,Fe_{2}B.$$

Considering Equations (2)–(5) and (23), the profiles of the Fe_2_B and FeB phases can be estimated as follows

(24) $$C_{Fe_{2}B}\left(x,t\right)=C_{int}^{Fe_{2}B}+\left(C_{low}^{Fe_{2}B}-C_{int}^{Fe_{2}B}\right)\left(\frac{erf\left(\frac{x}{2\sqrt{D_{Fe_{2}B}\left(t-t_{0}^{Fe_{2}B}\right)}}\right)-erf\left(\frac{u}{2\sqrt{D_{Fe_{2}B}\left(t-t_{0}^{Fe_{2}B}\right)}}\right)}{erf\left(\frac{v}{2\sqrt{D_{Fe_{2}B}\left(t-t_{0}^{Fe_{2}B}\right)}}\right)-erf\left(\frac{u}{2\sqrt{D_{Fe_{2}B}\left(t-t_{0}^{Fe_{2}B}\right)}}\right)}\right),$$

(25) $$C_{FeB}\left(x,t\right)=C_{up}^{FeB}+\frac{C_{low}^{FeB}-C_{up}^{FeB}}{erf\left(\frac{u}{2\sqrt{D_{FeB}\left(t-t_{0}^{FeB}\right)}}\right)}erf\left(\frac{x}{2\sqrt{D_{FeB}\left(t-t_{0}^{FeB}\right)}}\right).$$

#### 3.4.1. Phase Fe_2_B

For the mathematical formulation of the mass conservation equation in the diiron boride/substrate growth interface, consider the incoming flow with time dependence as: ${\vec{J}}_{Fe_{2}B}{\left(x,t\right)}_{x\;=\;v}\hat{i}=-D_{Fe_{2}B}{\left\{\partial C_{Fe_{2}B}\left(x,t\right)/\partial x\right\}}_{x\;=\;v}\hat{i}$ and the outflow also with time dependence: ${\vec{J}}_{Fe}{\left(x,t\right)}_{x\;=\;v+dv}\hat{i}=-D_{Fe}{\left\{\partial C_{Fe}\left(x,t\right)/\partial x\right\}}_{x\;=\;v+dv}\hat{i}=0$. Thus the equation can be written as follows:

(26) $$\left(\frac{C_{low}^{Fe_{2}B}-2C_{0}+C_{int}^{Fe_{2}B}}{2}\right){\left(\frac{dx}{dt}\right)|}_{x\;=\;v}\hat{i}=-D_{Fe_{2}B}{\frac{\partial C_{Fe_{2}B}\left(x,t\right)}{\partial x}|}_{x\;=\;v}\hat{i}.$$

Substituting Equations (10) and (24) in Equation (26), we obtain:

(27) $$\begin{array}{l} \left(\frac{C_{low}^{Fe_{2}B}-2C_{0}+C_{int}^{Fe_{2}B}}{2}\right)\varepsilon_{Fe_{2}B}=-\frac{\left(C_{low}^{Fe_{2}B}-C_{int}^{Fe_{2}B}\right)}{\sqrt{\pi }}\times \\ \times \left(\frac{exp\left(-\;\varepsilon_{Fe_{2}B}^{2}\right)}{erf\left(\frac{v}{2\sqrt{D_{Fe_{2}B}\left(t-\;t_{0}^{Fe_{2}B}\right)}}\right)\;-\;erf\left(\frac{u}{2\sqrt{D_{Fe_{2}B}\left(t-\;t_{0}^{Fe_{2}B}\right)}}\right)}\right). \end{array}$$

#### 3.4.2. Phase FeB

F For the mathematical formulation of the mass conservation equation in the iron monoboride/diiron boride growth interface, consider the incoming flow with time dependence as:

${\vec{J}}_{FeB}{\left(x,t\right)}_{x\;=\;v}\hat{i}=-D_{FeB}{\left\{\partial C_{FeB}\left(x,t\right)/\partial x\right\}}_{x\;=\;u}\hat{i}$ and the outflow also without time dependence as: ${\vec{J}}_{Fe_{2}B}{\left(x,t\right)}_{x\;=\;u+du}\hat{i}=-D_{Fe_{2}B}{\left\{\partial C_{Fe_{2}B}\left(x,t\right)/\partial x\right\}}_{x\;=\;u+du}\hat{i}.$ Thus the equation can be written as follows:

(28) $$\left(\frac{C_{up}^{FeB}-2C_{int}^{Fe_{2}B}+C_{low}^{FeB}}{2}\right){\left(\frac{dx}{dt}\right)|}_{x\;=\;u}\hat{i}=-D_{FeB}{\frac{\partial C_{FeB}\left(x,t\right)}{\partial x}|}_{x\;=\;u}\hat{i}-\left(-D_{Fe_{2}B}{\frac{\partial C_{Fe_{2}B}\left(x,t\right)}{\partial x}|}_{x\;=\;u+du}\right)\hat{i}$$

Replacing Equations (9), (24) and (25) into Equation (28), the following results:

(29) $$\begin{array}{l} \left(\frac{C_{up}^{FeB}-2C_{int}^{Fe_{2}B}+C_{low}^{FeB}}{2}\right)\left(\frac{\varepsilon_{FeB}D_{FeB}^{1/2}}{{\left(t-t_{0}^{FeB}\right)}^{1/2}}\right)\hat{i}=-D_{FeB}\frac{C_{low}^{FeB}-C_{up}^{FeB}}{\sqrt{\pi D_{FeB}\left(t-t_{0}^{FeB}\right)}erf\left(\frac{u}{2\sqrt{D_{FeB}\left(t-t_{0}^{FeB}\right)}}\right)}\times \\ \times \;exp\left(\frac{u^{2}}{4D_{FeB}\left(t-t_{0}^{FeB}\right)}\right)\;\hat{i}+\\ +\;D_{Fe_{2}B}\frac{C_{low}^{Fe_{2}B}-C_{int}^{Fe_{2}B}}{\sqrt{\pi D_{Fe_{2}B}\left(t-t_{0}^{Fe_{2}B}\right)}\left(erf\left(\frac{v}{2\sqrt{D_{Fe_{2}B}\left(t-t_{0}^{Fe_{2}B}\right)}}\right)-erf\left(\frac{u}{2\sqrt{D_{Fe_{2}B}\left(t-t_{0}^{Fe_{2}B}\right)}}\right)\right)}\times \\ \times \;exp\left(\frac{u^{2}}{4D_{Fe_{2}B}\left(t-t_{0}^{Fe_{2}B}\right)}\right)\;\hat{i}. \end{array}$$

## 4. Results

### 4.1. Microscopic Inspection of the Boride Coatings Formed on the Surface of the Ferrous Alloy

**Figure 8:**
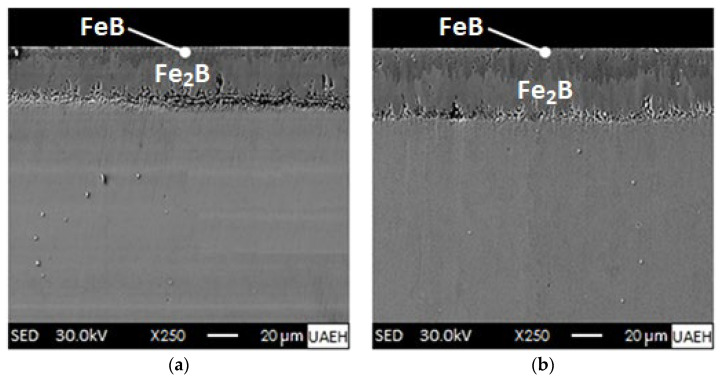

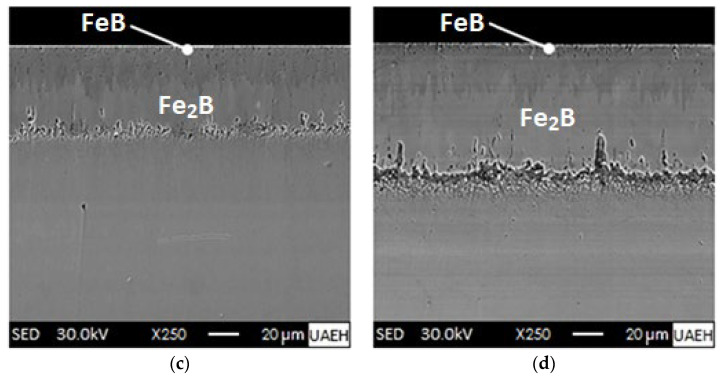
Microscopic examination by Scanning Electron Microscopy of AISI 420 stainless steel cross-sections exposed at 1273 K for varying times: (**a**) 2 h, (**b**) 4 h, (**c**) 6 h and (**d**) 8 h.

Looking at Figure 8, it is clear that the boride coatings on the AISI 420 steel surfaces have become more defined and extensive at a temperature of 1273 K with exposure times from 2 to 8 h. Further examination of the Scanning Electron Microscope images show the formation of a double phase consisting of iron monoboride as the external coating and underneath it the diiron boride coating (iron monoboride + diiron boride). Although the boride layers obtained are uniform and compact, they are not free of porosities. Boride layers are prone to generate high porosity at high treatment temperatures. The high degree of porosity in the boride layers implies a decrease in their mechanical properties because they represent stress concentration points [53]. The growth interfaces (iron monoboride/diiron boride and diiron boride/substrate) present a flat growth front due to the presence of chemical elements that make up the ferrous alloy (AISI 420 steel), mainly chromium, which is capable of forming chromium borides. The non-soluble alloying elements in the iron monoboride and diiron boride phases are expelled and pushed towards the core of the material and accumulate in the transition zone. This is a widespread occurrence in high alloy steels after boronizing [27,54,55,56,57]. The growth of the boride coatings is drastically affected by the alloying elements that make up AISI 420 steel, especially chromium (13.71%), and other chemical elements that form carbides such as niobium (0.02%), vanadium (0.09%), and titanium (0.005%). The depth of the double coating thicknesses (iron monoboride + diiron boride) ranges from 35.6 ± 8.9 to 74.4 ± 18.7 µm at 1273 K. It is noticeable that the thicknesses of the boride layers increase in relation to the treatment time for a selected temperature. In this sense, VillaVelázquez-Mendoza et al. [58] reported that the boronizing temperature has a more significant influence (about 67%) compared to the boronizing time (about 16%) on the dynamics of progression of the boronized coatings formed on the surface of an AISI 1018 steel (low-carbon steel with excellent weldability, considered as one of the best steels for boronizing, with a good balance of toughness, strength and ductility) applying the analysis of variance (ANOVA) technique also known as factor analysis, which is the essential tool for the study of the effect of one or more factors (each with two or more levels) on the measurement of a continuous variable.

### 4.2. X-ray Diffraction (XRD)

**Figure 9:**
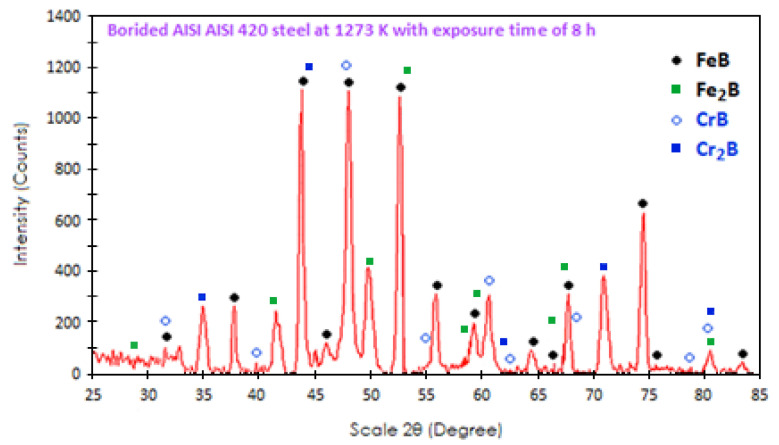
X-ray (XRD) diffraction pattern was generated from a hardened sample with a boronizing temperature of 1273 K with 8 h of exposure.

Figure 9 illustrates the most significant X-ray patterns (XRD) obtained on the surface of the boronized coatings at 1273 K for 8 h. The high chemical affinity of boron atoms with iron leads to the formation of iron borides (iron monoboride and diiron boride). In the particular case of chromium, it can form dispersed and independent chromium borides with a crystal lattice parameter very close to iron borides (iron monoboride and diiron boride) and dissolve in these phases. Due to the ability of chromium to concentrate in the outer part of the double phase (FeB–Fe_2_B) [59] the high chromium concentration in the substrate of about 13.7%, and the high chemical affinity of chromium with boron (chromium monoboride, chromium diboride, chromium tetraboride, chromium diboride, dichromium triboride, tetrachromium triboride, and pentachromium triboride) [25,60] coupled with the thickness at which the cobalt radiation used can penetrate ranges between 15 and 20 μm, the presence of chromium monoboride and chromium diboride phases in the X-ray analysis is explained. The identification of metal borides depends on their affinity for boron atoms and the selection of boronizing parameters and volume fractions. For example, Angkurarach and Juijerm [61] employed solid boronizing (powder-pack) supported by a continuous electromagnetic field to form boride coatings (iron monoboride and diiron boride) on stainless steel (AISI 420). The boronized specimen at the boronizing temperature of 1173 K with 4 h exposure time was subjected to basic X-ray diffraction (XRD) analysis, and only two iron borides, namely iron monoboride and diiron boride, were revealed. On the other hand, Turker Turkoglu and Ay [62] discovered, through XRD analysis, the presence of iron monoboride, diiron boride and chromium monoboride on the boron surface of the sample at 1273 K for 6 h. According to their research, they agree with our results.

### 4.3. Microhardness Vickers Profile

**Figure 10:**
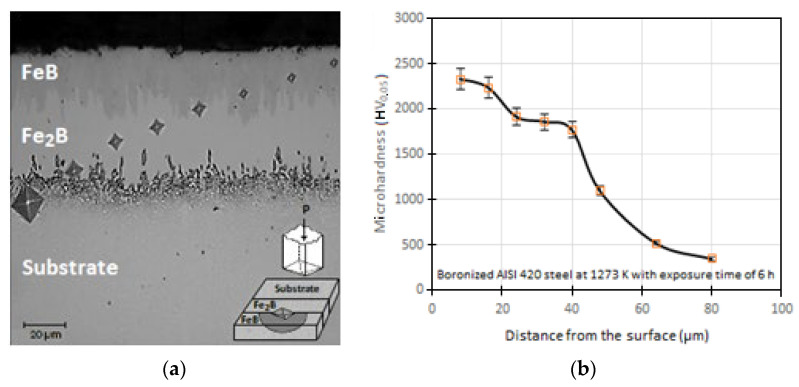
Representation of the cross-section of the boronized coating at the boronizing temperature of 1273 K with an exposure time of 6 h. (**a**): Microhardness traces made with a Vickers microhardness tester along the cross-section of the boronized coating, (**b**): Plot of the microhardness profile concerning the depth of the coating. The applied load was 4.903 × 10^−1^ Newtons.

Vickers microhardness tests evaluated the boronizing treatment and indicated that the surface hardening treatment was applied correctly. Figure 10a shows the cross-section of the boronized specimen at a temperature of 1273 K and 6 h of exposure, along with the microhardness profile performed using a Vickers indenter. Figure 10b presents the hardness values along the iron monoboride and diiron boride phases and the substrate (AISI 420 steel). It is evident that at 40 μm from the surface, the hardness values range from 2325 HV_0.05_ to 1767 HV_0.05_ for the hardened surface. In contrast, the untreated material has a hardness of approximately 345 HV_0.05_. The applied load was 4.903 × 10^−1^ Newton.

The Vickers indentations along the cross-section change in size continuously with the diffusion distance. The high hardness values directly result from the generation of iron borides, iron monoboride and diiron boride. The microhardness gradient of the microhardness Vickers profile follows the diffusion of boron atoms (boron atom flux) into the substrate. Turkoglu and Ay [62] carried out the application of the solid boronizing treatment on an AISI 420 stainless steel, where they performed a comparison of the Vickers microhardness profiles obtained along the cross-sections for a temperature of 1273 K with two treatment times of 4 h and 6 h. The Vickers microhardness value they found was approximately 1800 HV_0.05_ for 6 h, while for 4 h, the measured value was 1500 HV_0.05_. The boronizing time influenced the Vickers hardness profiles obtained. On the other hand, Gunes [63] applied a boronizing treatment to an AISI 420 stainless steel by solid diffusion, and found that the surface hardness was around 2150 HV_0.05_ for a temperature of 1223 K and exposure time of 5 h, which is congruent with our findings. Likewise, Kayali and Taktak [57] carried out a boronizing treatment on an AISI 420 stainless steel for a temperature of 1123 K for 4 h exposure and found that from 10 μm from the cross-sectional surface of the boronized coating, the Vickers microhardness takes a value of about 1972 ± 4 HV_0.05_, which is very close to that reported in our research. In another work, the surface hardness values measured by Kayali [64] were in the interval 1779–1972 HV_0.05_ for the AISI 420 steels treated at 1123 K, 1173 K, and 1223 K, while the hardening of untreated substrate attained a value of 340 HV_0.05_. Considering other works, Kayali [64] applied boronizing treatment to AISI 304 steels, subject to temperatures of 1123 K, 1173 K and 1223 K, reported that the hardness reached by the boronized coatings was 1640 –1904 HV_0.05_. On the other hand, the Vickers hardness acquired by the untreated substrate was approximately 225 HV_0.05_. In other research, Kayali [64] measured the surface hardness values of AISI 304L steels treated at 1123 K, 1173 K, and 1223 K. The values fell within the range of 1778 –2074 HV_0.05_. On the other hand, the untreated substrate had a hardness of only 229 HV_0.05_.

### 4.4. Cohesion by Daimler–Benz Rockwell-C Test

After forming the boronized coatings on the surface of AISI 420 stainless steel, their cohesive nature was evaluated using the Daimler–Benz Rockwell-C indentation technique. For this purpose, two specimens hardened under the following conditions were considered: 1123 K at 2 h and 1273 at 8 h. Scanning Electron Microscopy (SEM) images of the obtained craters were compared with the standard patterns established in VDI 3198 [46].

**Figure 11:**
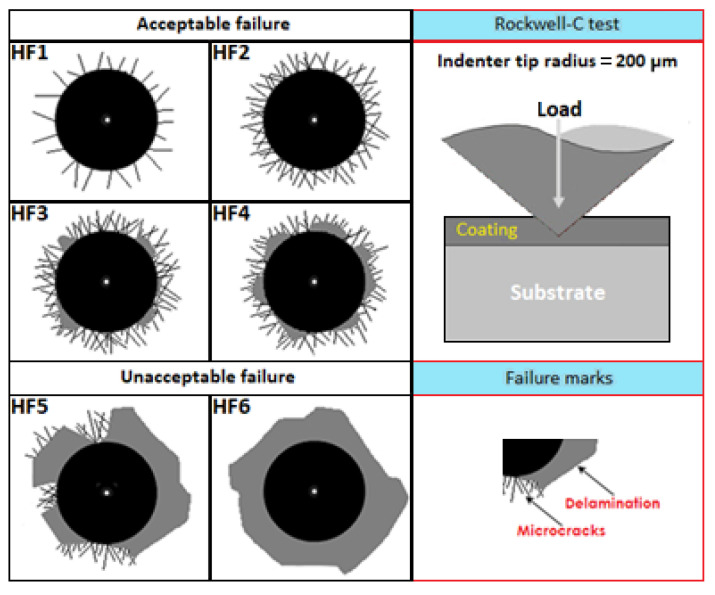
The comparative image pattern has been used to evaluate the adhesion quality of the boronized coatings during the Daimler–Benz Rockwell-C technique. HF1–HF4 represent the quality maps of adhesion strength; generally, there is sufficient adhesion. HF5 or HF6 represent insufficient adhesion.

**Figure 12:**
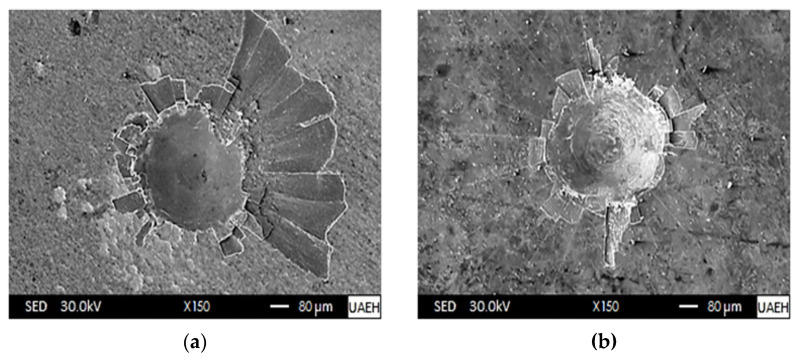
Scanning Electron Microscopy (SEM) images of the craters generated by the Daimler–Benz Rockwell-C indenter on the surface of the boronized coatings for two different times and temperatures (**a**): 1273 K at 8 h, (**b**): 1123 K at 2 h.

According to the patterns in Figure 11, the categories ranging from HF1 to HF4 are considered acceptable quality, unlike those from HF5 to HF6, which are undesirable due to poor cohesive strength on the substrate [65,66]. The indentations generated by the Daimler–Benz Rockwell-C technique on the surfaces of the boronized coatings are shown in Figure 12; in the images obtained by Scanning Electron Microscopy (SEM), it can be seen that radial cracks arise in both specimens around the perimeter of the craters created by the Daimler–Benz Rockwell-C technique.

As we observe in Figure 12a, the cracks resulting from the Daimler–Benz Rockwell-C indenter on the hardened surface at 1273 K with 8 h of exposure extend radially, accompanied by an apparent structural failure caused by delamination around the generated crater by the application of a mechanical stress. Contrastingly, Figure 12b displays significantly less structural damage with a boronizing temperature set at 1123 K for 2 h. Based on the research findings, boronizing at a temperature of 1273 K with an 8-hour exposure generates a coating thickness greater than boronizing at a temperature of 1123 K with a 2-hour exposure. However, the adhesion quality of the coating thickness falls under the HF5 category, which is considered objectionable according to the adhesion quality patterns in Figure 11. This is because the thicker boronized coating is susceptible to generating higher tensile residual stresses. It is fascinating to see how something as small as an indentation can significantly impact the material’s mechanical properties. It shows that compressive stresses are produced just below the indentation, but tensile stresses are induced at the edge of the indentation [66]. After examining the boronized coating produced at a temperature of 1123 K with an exposure time of 2 h, it is clear that it boasts a superior adhesion quality. This can be linked to the HF4 category pattern in Figure 11, which is deemed satisfactory. Kayali and Taktak [57] applied the thermochemical boronizing treatment to AISI 420 stainless steel to study the cohesion of the boronized coatings (iron monoboride and diiron boride), which were produced at the temperatures of 1123 K and 1223 for a single exposure time of 4 h. In their findings, they highlighted that the coatings produced at the temperature of 1123 K presented a better adhesion quality which, according to Figure 11, can be related to the HF2–HF3 categories, while the boronized coating generated at the temperature of 1223 K with 4 h was characterized by a poor surface adhesion which was related to the HF5 pattern.

### 4.5. Pin-on-Disc Testing

**Figure 13:**
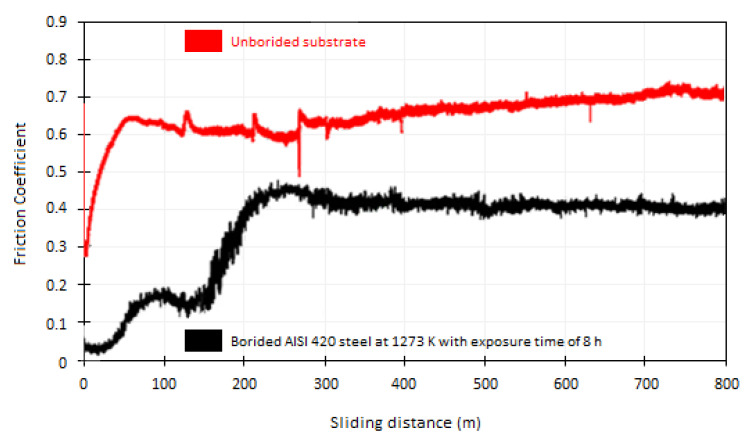
Performance of the coefficient of friction concerning the relative sliding distance of unboronized and boronized AISI 420 stainless steel surface.

In pin-on-disc tests, our objective was to evaluate the wear behaviour of boronized coatings (iron monoboride and diiron boride) bonded to stainless steel AISI 420. We focused on the study of tribology, which involves the study of friction, wear, and lubrication. We performed the tests under non-lubricated conditions and observed that the frictional force changed steadily with sliding distance. We recorded the variation of the coefficient of friction as a function of sliding distance to analyze the tribological behaviour of boron-free and boron-containing surfaces. These data will help us better understand the wear characteristics of these coatings and develop more efficient materials for tribological applications. Figure 13 presents two studies on the behaviour of the friction coefficient concerning the sliding distance on the boron-free surface (substrate–stainless steel AISI 420) and with boron (iron monoboride and diiron boride coatings). For the boronized surface produced at the temperature of 1273 K with an exposure time of 8 h, the coefficient of friction increases in magnitude for the 0 to 100 m and 150 to 250 m intervals resulting in the shearing-off of roughness during the break-in period. Meanwhile, in the intervals from 250 to 500 m, it gradually decreases until it reaches a constant value of approximately 0.4 from 500 m (see Figure 13). These findings suggest that the surface treatment process has a significant impact on the performance and durability of materials. It is important to carefully consider the effects of temperature and time on the surface properties of materials in order to optimize their performance and extend their lifespan.

**Figure 14:**
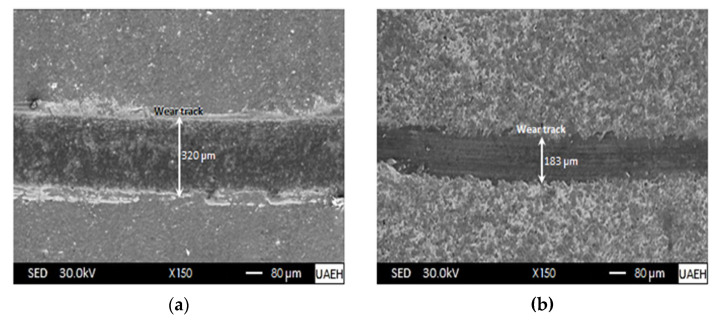
Wear tracks obtained by Scanning Electron Microscopy (SEM): (**a**) boron-free surface (AISI 420 stainless steel) and (**b**) hardened surface through boronizing treatment considering a temperature of 1273 K and an exposure time of 8 h.

Analyzing the behaviour of the friction coefficient of the untreated sample, i.e., free of boron, from the intervals of 0 m and 50 m, we have a positive slope of about 7 × 10^–3^ m^−1^, where the flattening of most of the debris detached from the boronized coating takes place. Subsequently, the value of the friction coefficient decreases gradually up to a distance of approximately 130 m, maintaining a constant behaviour up to a distance of 250 m, and continuing with a smooth increase until reaching a value of 0.7, as shown in Figure 13. With these results, we can place the friction coefficient for the boronized surface between the values of 0.05 and 0.4, and for the boron-free surface (AISI 420 stainless steel), the friction coefficient is between the values of 0.3 and 0.7. It can be appreciated that the wear resistance of AISI 420 steel can be significantly improved by treating it with boronizing coatings. This treatment reduces the friction coefficient on the steel’s surface, enhancing its durability. It was found that stable carbides can act as solid lubricants inside boride coatings, which can significantly decrease frictional forces [67,68]. This has been observed in other studies as well and is an important factor to consider when looking at the performance of boride coatings [67,68]. Gunes [63] conducted tribological characterization of the hardened surface of an AISI 420 stainless steel with the ball-on-disc configuration through the thermochemical boronizing process. The sliding distance was 1000 m, at a constant velocity of 0.3 m/s. All tests were carried out under dry conditions, i.e., without lubrication. According to the results obtained, the value of the wear rate for AISI 420 stainless steel hardened with the boronizing thermochemical treatment at a temperature of 1223 K with an exposure time of 5 h was 2 × 10^–5^ mm^3^∙N^−1^m^−1^, while for the boron-free substrate, i.e., without treatment, the value of the wear rate was 4 × 10^–5^ mm^3^∙N^−1^m^−1^. It was discovered that the boronizing process significantly enhances the tribological properties of stainless steel AISI 420 that has been boronized. Figure 14 shows the wear tracks corresponding to the boronized surface at a temperature of 1273 K with an exposure time of 8 h and the boron-free surface, i.e., untreated, both obtained by Scanning Electron Microscopy (SEM). It can be observed in Figure 14a, which corresponds to the untreated surface, that the width of the wear track is about 320 μm, which is much larger than the wear track for the hardened surface, which is approximately 183 μm. Both wear tracks show scratch lines, wear debris, and plastic deformation. Figure 14a, corresponding to the untreated specimen (AISI 420 stainless steel), shows the transfer of material from one surface to another due to the predominantly adhesive wear. On the other hand, due to the presence of the boronized coatings (iron monoboride and diiron boride) and the dispersed carbides, the width of the wear track is significantly reduced, as can be seen in Figure 14b. In addition, because the hardened surface (boronized surface) slides on a considerably softer surface, there is wear of an abrasive nature. It is noteworthy that although the surface of AISI 420 stainless steel is free of boron, i.e., without treatment, it shows a cautious wear behaviour compared to the specimens hardened through boronizing treatment for low-carbon steel (AISI 1018) [69]; this phenomenon can be explained because the AISI 420 martensitic stainless steel is characterized by the presence of chromium, which is the primary alloying element (13.71% Chromium). In the microstructure without any heat treatment, one can observe ferrite grains and chromium carbides that form during the solidification process, which ultimately help the surface subjected to wear as they behave as lubricants and consequently decrease the wear rate.

### 4.6. Determination of Parabolic Growth Law Constants of Boronized Coatings (Iron Monoboride and Diiron Boride)

**Figure 15:**
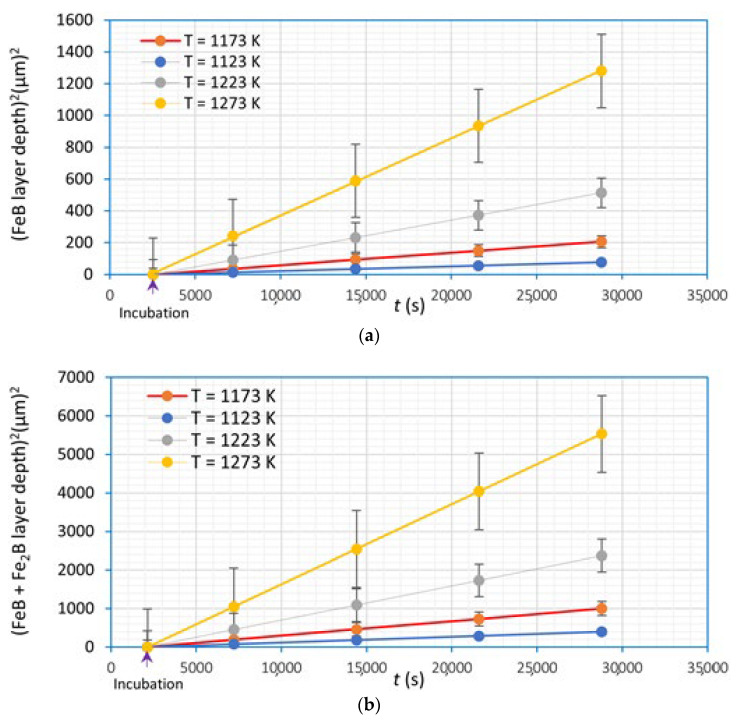
Graph of the squared thickness of the boronized coatings versus exposure time for each boronizing temperature: (**a**) iron monoboride coating, (**b**) total coating (iron monoboride + diiron boride).

Figure 15 plots the squared thickness of the iron monoboride coating versus exposure time in the same way for the total coating (iron monoboride + diiron boride). From the slopes of the plotted curves, constants were obtained that integrate the parabolic growth laws, which model the growth of the boronized coatings (see Equations (9) and (10)) in the selected range of boronizing temperatures. Table 1 summarizes each phase’s temperatures, the constants of the parabolic growth laws, and the incubation time corresponding to each phase. 

#### 4.6.1. Determination of Diffusion Coefficients of Boronized Coatings (Iron Monoboride and Diiron Boride) by the First Mass Transfer Model without Time Dependence

Substituting the concentration values in Equations (14) and (21), the constants $\varepsilon_{FeB}^{2}$ and $\varepsilon_{Fe_{2}B}^{2}$ are estimated and clustered in Table 2 for the highest amount of boron concentration (B) in iron monoboride corresponding to the value of 16.4 wt.% and diiron boride corresponding to the value of 9 wt.%.

From Table 2, the diffusion coefficients of boron in FeB and Fe_2_B ($D_{FeB}$ and $D_{Fe_{2}B}$) have been obtained. Table 3 shows the values for each diffusion coefficient concerning the treatment temperature.

**Figure 16:**
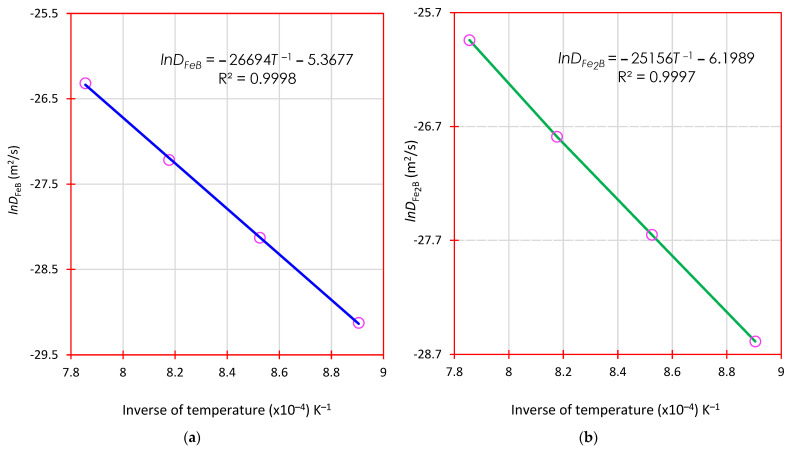
The plot of the natural logarithm of the diffusion coefficient versus the inverse of boronizing temperature: (**a**) The blue slope corresponds to the activation energy of the monoboride iron and (**b**) The green slope corresponds to the activation energy of the diiron boride through the first mass transfer model.

Figure 16 plots lnD against the inverse of boronizing temperature (1/*T*); an Arrhenius-type relationship ($D_{i}=D_{0}^{i}exp(-Q_{i}/RT)\;with=FeB,Fe_{2}B$) was assumed according to the data obtained in Table 3.

From the experimental data plotted in Figure 16, mathematical expressions have been derived by linear regression for each diffusion coefficient in the boronized phases (iron monoboride and diiron boride) as follows:

(30) $$D_{FeB}=4.6\times 10^{-3}exp\left(-\frac{222.9\;\mathrm{kJ}\cdot {\mathrm{mol}}^{-1}}{RT}\right),$$

(31) $$D_{Fe_{2}B}=2.0\times 10^{-3}exp\left(-\frac{209.1\;\mathrm{kJ}\cdot {\mathrm{mol}}^{-1}}{RT}\right).$$

where *R* = 8.3 J/mol∙K and *T* is the boronizing temperature in Kelvin. 

#### 4.6.2. Determination of Diffusion Coefficients of Boronized Coatings (Iron Monoboride and Diiron Boride) by the Second Mass Transfer Model with Time Dependence

A MATLAB program was developed to determine the constants $\varepsilon_{FeB}^{2}$ and $\varepsilon_{Fe_{2}B}^{2}$ considering Equations (27) and (29) using the Newton–Raphson method [70] and clustered in Table 4 for the highest amount of boron concentration (B) in iron monoboride corresponding to the value of 16.4 wt.% and diiron boride corresponding to the value of 9 wt.%.

From Table 4, the boron diffusion coefficients for each phase ($D_{FeB}$ and $D_{Fe_{2}B}$) were obtained. Table 5 shows the values for each diffusion coefficient concerning the treatment temperature.

**Figure 17:**
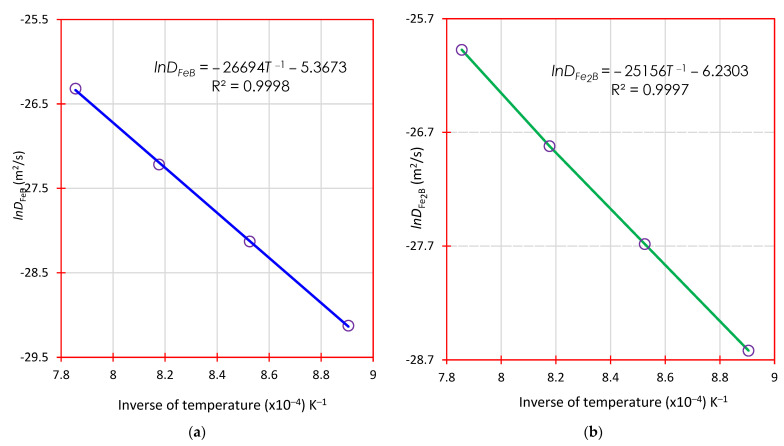
The plot of the natural logarithm of the diffusion coefficient versus the inverse of boronizing temperature: (**a**) The blue slope corresponds to the activation energy of the monoboride iron and (**b**) The green slope corresponds to the activation energy of the diiron boride through the second mass transfer model.

Figure 17 plots lnD against the inverse of boronizing temperature (1/*T*); an Arrhenius-type relationship ($D_{i}=D_{0}^{i}exp(-Q_{i}/RT)\;with=FeB,Fe_{2}B$) was assumed according to the data obtained in Table 5.

From the experimental data plotted in Figure 17, mathematical expressions have been derived by linear regression for each diffusion coefficient in the boronized phases (iron monoboride and diiron boride) as follows:

(32) $$D_{FeB}=4.6\times 10^{-1}exp\left(-\frac{221.9\;\mathrm{kJ}\cdot {\mathrm{mol}}^{-1}}{RT}\right),$$

(33) $$D_{Fe_{2}B}=1.9\times 10^{-3}exp\left(-\frac{209.1\;\mathrm{kJ}\cdot {\mathrm{mol}}^{-1}}{RT}\right).$$

where *R* = 8.3 J/mol∙K and *T* is the boronizing temperature in Kelvin. 

## 5. Discussion

### 5.1. Discussion of the Two Diffusion Models

The values obtained for $\varepsilon_{FeB}^{2}$ and $\varepsilon_{Fe_{2}B}^{2}$ for both mathematical diffusion models are equivalent (see Table 2 (steady-state diffusion model) and Table 4 (diffusion model with time dependence)). To understand this equivalence, we will start with the FeB phase. If we develop in the Taylor series [71] the functions that integrate Equation (29) and considering only the first order of the series, we obtain:

(34) $$\begin{array}{l} \left(\frac{C_{up}^{FeB}-2C_{int}^{Fe_{2}B}+C_{low}^{FeB}}{2}\right)\left(\frac{\varepsilon_{FeB}D_{FeB}^{1/2}}{{\left(t-t_{0}^{FeB}\right)}^{1/2}}\right)\hat{i}=-D_{FeB}\frac{C_{low}^{FeB}-C_{up}^{FeB}}{\frac{2}{\sqrt{\pi }}\sqrt{\pi D_{FeB}\left(t-t_{0}^{FeB}\right)}\left(\frac{u}{2\sqrt{D_{FeB}\left(t-t_{0}^{FeB}\right)}}\right)}\hat{i}\\ +\;D_{Fe_{2}B}\frac{C_{low}^{Fe_{2}B}-C_{int}^{Fe_{2}B}}{\frac{2}{\sqrt{\pi }}\sqrt{\pi D_{Fe_{2}B}\left(t-t_{0}^{Fe_{2}B}\right)}\left(\frac{v}{2\sqrt{D_{Fe_{2}B}\left(t-t_{0}^{Fe_{2}B}\right)}}-\frac{u}{2\sqrt{D_{Fe_{2}B}\left(t-t_{0}^{Fe_{2}B}\right)}}\right)}\hat{i}. \end{array}$$

Rewriting Equation (34) and combining it with Equation (12), we arrive at the following:

(35) $$\left(\frac{C_{up}^{FeB}-2C_{int}^{Fe_{2}B}+C_{low}^{FeB}}{2}\right)\left(2\varepsilon_{FeB}D_{FeB}^{1/2}\right)\hat{i}=-D_{FeB}\left(\frac{C_{low}^{FeB}-C_{up}^{FeB}}{\varepsilon_{FeB}D_{FeB}^{1/2}}\right)\hat{i}+D_{Fe_{2}B}\left(\frac{C_{int}^{Fe_{2}B}-C_{up}^{Fe_{2}B}}{\varepsilon_{FeB}D_{FeB}^{1/2}}\right)\hat{i}.$$

Equation (35) is the same as Equation (18). Continuing the analysis of the Fe_2_B phase. We develop in the Taylor series the functions that integrate Equation (27)

(36) $$\left(\frac{C_{low}^{Fe_{2}B}-2C_{0}+C_{int}^{Fe_{2}B}}{2}\right)\varepsilon_{Fe_{2}B}=-\frac{\left(C_{low}^{Fe_{2}B}-C_{int}^{Fe_{2}B}\right)}{\sqrt{\pi }}\left(\frac{1}{\frac{2}{\sqrt{\pi }}\left(\frac{1}{2\sqrt{D_{Fe_{2}B}\left(t-\;t_{0}^{Fe_{2}B}\right)}}\right)\;\left(v-u\right)}\right).$$

Rewriting Equation (36) and combining it with Equation (12), we arrive at the following:

(37) $$\left(\frac{C_{low}^{Fe_{2}B}-2C_{0}+C_{int}^{Fe_{2}B}}{2}\right)\frac{\varepsilon_{Fe_{2}B}}{{\left(t-t_{0}^{Fe_{2}B}\right)}^{1/2}}=-D_{Fe_{2}B}^{1/2}\left(\frac{C_{low}^{Fe_{2}B}-C_{up}^{Fe_{2}B}}{v}\right).$$

Equation (37) is precisely the same as Equation (13).

### 5.2. Derivation of the Activation Energies of Boron in FeB and Fe_2_B: Comparison with the Literature

As we can see from Table 6, there are significant differences between the values of boron activation energies in iron borides obtained from the literature and those estimated in our study [55,56,62,64,72,73,74,75]. This can be attributed to several factors, including the boronizing method used, the approach employed for assessing the activation energies, the boronizing parameters chosen, the chemical compositions of the steels, and the types of heating used to achieve the boronizing treatment. These factors can significantly impact the results obtained, and careful consideration must be given to them when conducting similar studies in the future. As we can see from the data in Table 6, there are notable variations and resemblances in the values of diffusion coefficients; Keddam et al. [49] applied paste-boronizing treatment, where the boron donor source was borax; the temperature range was from 973 K to 1073 K to harden AISI 440C steel. They found that the estimated boron activation energy value (134.6 kJ∙mol^−1^) was slightly lower than that reported in other works [56,62,64,72,73,74,75]. Ayvaz and Aydin [56] hardened medical-grade stainless steel (AISI 316L) using boronizing treatment and studied the effects of microwave heating on the dynamics of the boronized coatings. Their findings determined the value of the minimum energy (Q = 244.1 kJ∙mol^−1^) through microwave heating. Likewise, they revealed that the growth of boronized coatings could increase by a factor of two when using microwave heating instead of conventional heating methods like an oven. In another interesting study, Kayali and Mertgenç [72] applied the thermochemical boronizing treatment to an AISI 303 stainless steel in a temperature range from 1173 K to 1223 K. Their findings showed that the minimum energy of iron borides is around 236.5 kJ∙mol^−1^, which is consistent with other reported studies [55,62,75]. In other research, Kayali [64] hardened different stainless steels (AISI 420, AISI 304 and AISI 304L) by thermochemical boronizing treatment employing the powder-pack technique in the temperature range from 1123 K to 1223 K, and, considering that the boronized coatings obey the parabolic growth law, was able to determine the minimum energies for each steel (Q_AISI 420_ = 206.1 kJ∙mol^−1^, Q_AISI 304_ = 234.6 kJ∙mol^−1^ and, Q_AISI 304L_ = 222.8 kJ∙mol^−1^). Juijerm [73] successfully hardened AISI 420 stainless steel by using two different boronizing methods. He utilized salt baths and powder-pack methods within a temperature range of 1123 K to 1223 K. Through his research, Juijerm discovered that the boronizing process directly impacts the minimum energy estimation. He found that the salt bath method consumes more energy than the powder-pack method. These findings provide a valuable insight into the most efficient and effective ways of hardening steel. Turkoglu and Ay [62] hardened three stainless steels (AISI 420, AISI 304 and AISI 430) through the thermochemical treatment of solid boronizing in the temperature range from 1123 K to 1198 K. They estimated the value of the minimum energies for each steel: Q_AISI 420_ = 242.1 kJ∙mol^−1^, Q_AISI 304_ = 182.4 kJ∙mol^−1^ and, Q_AISI 430_ = 151.4 kJ∙mol^−1^. AISI 430 steel should have a significantly higher activation energy value, as reported by other authors [56,62,72,75]. It is essential to consider all factors when analyzing the properties of different types of steel. In an interesting article by Campos-Silva et al. [74], they applied a unique thermochemical boronizing treatment to medical-grade stainless steel (AISI 316L). They used the pulsed direct current-assisted powder-pack technique, which decreased the minimum boron energies compared to conventional boronizing methods [56,62,64,72,75]. Topuz et al. [75] hardened AISI 304 stainless steel by the powder-pack boronizing method, using a very particular heating technique known as “indirect heating of a fluidized bed”, which ensures uniform heating of the thermochemical process and homogeneous diffusion of boron atoms on the surface of the substrate. To determine the minimum energy, they used the parabolic growth law for the coating formed by the iron monoboride and diiron boride, resulting in Q_AISI 304_ = 244 kJ∙mol^−1^. Our findings on AISI 420 stainless steel activation energies align with previous research in this field. It gives us confidence in the accuracy of our results and allows us to make more informed decisions moving forward [62,64].

### 5.3. Verification of the Mathematical Models of Mass Transfer by Experimental Data

**Figure 18:**
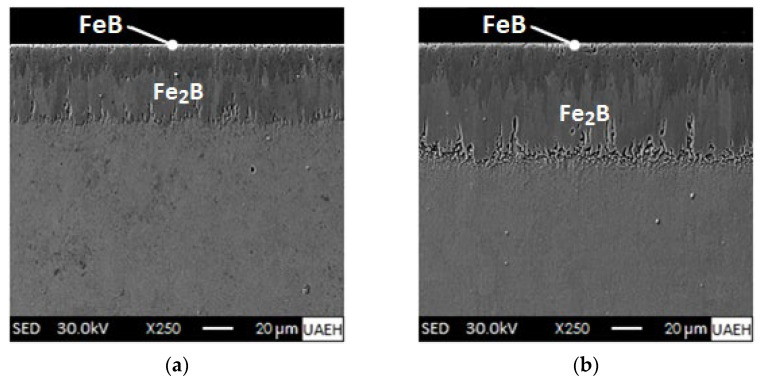
Microscopic inspection by Scanning Electron Microscopy (SEM) of AISI 420 stainless steel cross-sections treated with a unique time of 5.5 h: (**a**) 1273 K and (**b**) 1323 K.

According to Table 7, the experimental and simulated thicknesses of the (iron monoboride, $u_{sim}$) and (iron monoboride + diiron boride, $v_{sim}$) coatings were recorded for two specific boronizing conditions. These findings could be useful for further research in the field of boronizing surface treatments. Figure 18 shows the cross-sections of the boronized coatings produced on the surface of AISI 420 stainless steel by Scanning Electron Microscopy (SEM) considering two boronizing temperatures of 1273 K and 1323 K, respectively, during a single exposure time of 5.5 h. Likewise, based on our analysis of the experimental data, the thickness of the boronized coating is consistent with what the mathematical diffusion model predicted. This is certainly promising news, but we must also recognize that our findings have certain limitations; only one-way diffusion of boron was considered, and the interaction between carbon (C) and silicon (Si) atoms with boron (B) atoms during mass flow was not taken into account.

As complementary information to our work, it is necessary to emphasize that the phenomenon of precipitation of metallic borides inside the boronized coating was neglected. However, Anantha et al. [76] found in their optical microscopy and SEM–EDX analysis that a substantial proportion of precipitates were uniformly dispersed throughout the microstructure of the steel, and some appeared to be adhered to the grain boundaries. Interestingly, the carbide particles they found were rounded in shape, and some exhibited spherical and capsular configurations. In their Energy Dispersive X-ray (EDX) analysis of a specific zone, they found that it contained particles that did not dissolve and showed a notable concentration of chromium and carbon due to a decrease in iron, a sign of chromium-rich carbides. They did not observe any other enrichment or depletion of alloying elements.

Determining the diffusion coefficients ($D_{i}=D_{0}^{i}exp(-Q_{i}/RT)\;with=FeB,Fe_{2}B$) of the iron monoboride and diiron boride phases allows the estimation of the layer thicknesses through the expressions given for *u* (FeB) and v (Fe_2_B), which is very important in industrial applications. The service conditions of thick boride layers are recommended for work with erosion (abrasive wear), such as extruding plastic materials loaded with glass fiber or pigments, such as titanium oxide. For applications where the boride layers are subjected to erosion–corrosion, it is suggested to use relatively thick coatings, from 30 to 70 μm, producing excellent layer/substrate adhesion [3].

## 6. Conclusions

The study showed that the surface properties of AISI 420 stainless steel were significantly improved by powder-pack boronizing treatment. The treatment was carried out in the temperature range of 1123 K to 1273 K with exposure times of 2 h, 4 h, 6 h and 8 h. The boride coatings formed during the process improved the material’s metallurgical, mechanical and tribological properties. This treatment is, therefore, an effective measure to increase the durability and wear resistance of AISI 420 steel. The findings are summarized in the following points:

- XRD analysis verified the presence of iron monoboride, diiron boride, chromium monoboride, and chromium diiron boride phases. Scanning Electron Microscopy (SEM) images of the cross-sections of the boronized coatings showed that the growth fronts tend to be flat.
- The surface hardness profile of AISI 420 stainless steel hardened by thermochemical boronizing treatment reached elevated hardnesses from about 1767 HV_0.05_ to 2250 HV_0.05_ at 40 μm from the surface and 345 HV_0.05_ in the core of the untreated material.
- Based on the data obtained from the Daimler-Benz Rockwell-C indentations of the adhesion tests of the boronized coatings, it was found that the specimen hardened at a temperature of 1123 K for 2 h, exhibited adhesion quality that fell under the HF4 category, as shown in Figure 11 of the comparative patterns. This indicates that the specimen has sufficient cohesion quality. On the other hand, the sample hardened at a temperature of 1273 K for 8 h is associated with the HF5 category, meaning it does not have sufficient adhesion.
- The values of the friction coefficients for the untreated sample ranged from 0.3 to 0.7. On the other hand, for the hardened specimen at a temperature of 1273 K with 8 h of exposure time, the values ranged from 0.05 to 0.4. It is interesting how the treatment affected the range of values.
- The results of the pin-on-disc tests showed that the untreated specimen (AISI 420 stainless steel) showed greater predominantly adhesive wear. On the other hand, due to the presence of the boronized coatings (iron monoboride and diiron boride) and the dispersed carbides acting as solid lubricants, the width of the wear track was significantly reduced, improving the wear resistance considerably, as can be seen in Figure 14b. In addition, as the hardened surface (boronized surface) was sliding on a much softer surface, wear of an abrasive nature occurred.
- Very similar results were obtained for the constants $\varepsilon_{FeB}^{2}$ and $\varepsilon_{Fe_{2}B}^{2}$ for both proposed mathematical diffusion models (model without time dependence and with time dependence); this equivalence was demonstrated through the Taylor series development of the functions that integrate Equations (27) and (29) which refer to the diffusion model with time dependence, considering only the first order of the series, which allows for reproducing the results of the model without time dependence (see Equations (14) and (21)).
- After conducting experiments on AISI 420 stainless steel, it was found that the mathematical mass transfer models used were equivalent. The activation energies for the iron monoboride and diiron boride phases formed on the surface were also estimated. The values obtained were $Q_{FeB}=$ 221.9 kJ∙mol^−1^ and $Q_{Fe_{2}B}=$ 209.1 kJ∙mol^−1^. These values were compared with those found in the literature, which can be found in Table 6.
- After performing experiments for two different boronizing conditions, we verified the steady-state mass transfer model. The results obtained agree satisfactorily with the simulations (see Table 7). This shows that the model is accurate and can be relied upon for future experiments.

## Figures and Tables

**Table 1:** Summarizes the boronizing treatment temperatures (1123 K, 1173 K, 1223 K, and 1273 K), parabolic growth law constants and incubation time corresponding to each phase.

Experimental Temperature T (K)	Experimental Constant $2\varepsilon_{FeB}D_{FeB}^{1/2}$ (µm/s^1/2^)	Incubation Time $t_{0}^{FeB}$ (s)	Experimental Constant $2\varepsilon_{Fe_{2}B}D_{Fe_{2}B}^{1/2}$ (µm/s^1/2^)	Incubation Time $t_{0}^{Fe_{2}B}$ (s)
1123	0.05	2528	0.12	2124
1173	0.08	2539	0.19	2135
1223	0.14	2535	0.29	2128
1273	0.22	2536	0.45	2136

**Table 2:** Dimensionless constants $\varepsilon_{FeB}^{2}$ and $\varepsilon_{Fe_{2}B}^{2}$ were obtained from Equations (14) and (21), respectively (Steady-State Diffusion Model).

Experimental Temperature T (K)	Constant $\varepsilon_{FeB}^{2}$ (without Units)	Experimental Constant $2\varepsilon_{FeB}D_{FeB}^{1/2}$ (µm/s^1/2^)	Constant $\varepsilon_{Fe_{2}B}^{2}$ (without Units)	Experimental Constant $2\varepsilon_{Fe_{2}B}D_{Fe_{2}B}^{1/2}$ (µm/s^1/2^)
1123	3.2 × 10^–3^	0.05	9.6 × 10^–3^	0.12
1173		0.08		0.19
1223		0.14		0.29
1273		0.22		0.45

**Table 3:** Diffusion coefficients generated by the Steady-State Diffusion Model.

Experimental Temperature T (K)	Boron Diffusivity in FeB $D_{FeB}$ (m^2^/s)	Boron Diffusivity in Fe_2_B $D_{Fe_{2}B}$ (m^2^/s)
1123	2.2 × 10^−13^	3.8 × 10^−13^
1173	6.1 × 10^−13^	9.8 × 10^−13^
1223	1.5 × 10^−12^	2.310^−12^
1273	3.7 × 10^−12^	5.4 × 10^−12^

**Table 4:** Values of constants $\varepsilon_{FeB}^{2}$ and $\varepsilon_{Fe_{2}B}^{2}$ were obtained from Equations (27) and (29), respectively (Diffusion model with time dependence).

Experimental Temperature T (K)	Constant $\varepsilon_{FeB}^{2}$ (without Units)	Experimental Constant $2\varepsilon_{FeB}D_{FeB}^{1/2}$ (µm/s^1/2^)	Constant $\varepsilon_{Fe_{2}B}^{2}$ (without Units)	Experimental Constant $2\varepsilon_{Fe_{2}B}D_{Fe_{2}B}^{1/2}$ (µm/s^1/2^)
1123	3.2 × 10^–3^	0.05	9.8 × 10^–3^	0.12
1173		0.08		0.19
1223		0.14		0.29
1273		0.22		0.45

**Table 5:** Diffusion coefficients generated by the diffusion model with time dependence.

Experimental Temperature T (K)	Boron Diffusivity in FeB $D_{FeB}$ (m^2^s)	Boron Diffusivity in Fe_2_B $D_{Fe_{2}B}$ (m^2^s)
1123	2.2 × 10^−13^	3.7 × 10^−13^
1173	6.1 × 10^−13^	9.5 × 10^−13^
1223	1.5 × 10^−12^	2.2 × 10^−12^
1273	3.7 × 10^−12^	5.2 × 10^−12^

**Table 6:** Documented interesting findings on boron’s minimum energies (Q) in high alloy steels.

Substrate	Thermochemical Treatment	Activation Energies (kJ∙mol−1)	Temperature Interval of Investigation (K)	Growth Law	Refs.
AISI 4040 C	Plasma-paste	(FeB + Fe_2_B) 134.6	973–1073	Parabolic	[55]
AISI 316L	Powder with microwave heating	(FeB + Fe_2_B) 244.1	1073–1223	Parabolic	[56]
AISI 303	Powder	(FeB + Fe_2_B) 236.5	1123–1223	Parabolic	[72]
AISI 420	Powder	(FeB + Fe_2_B) 206.2	1123–1223	Parabolic	[64]
AISI 420	Salt-bath	(FeB + Fe_2_B) 233.5	1123–1223	Parabolic	[73]
AISI 420	Powder	(FeB + Fe_2_B) 185.2	1123–1223	Parabolic	[73]
AISI 420	Powder	(FeB + Fe_2_B) 242.1	1123–1273	Parabolic	[62]
AISI 430	Powder	(FeB + Fe_2_B) 151.4	1123–1273	Parabolic	[62]
AISI 316L	Pulsed-DC powder-pack boronizing	(FeB) 162, (Fe_2_B) 171	1123–1223	Bilayer model	[74]
AISI 304	Powder with indirect heating in fluidized bed	(FeB + Fe_2_B) 244	1123–1323	Parabolic	[75]
AISI 420	Powder	(FeB) 221.9, (Fe_2_B) 209.1	1123–1273	Parabolic	This work

**Table 7:** Comparison of the coating thicknesses obtained by the boronizing thermochemical treatment considering two treatment temperatures and a single exposure time and the coating thicknesses estimated from Equations (9) and (10).

Boronizing Conditions	Experimental Layer Thickness $u_{exp}$ (µm)	Simulated Layer Thickness $u_{sim}$ (µm) Equation (9)	Experimental Layer Thickness $v_{exp}$ (µm)	Simulated Layer Thickness $v_{sim}$ (µm) Equation (10)
1273 K for 5.5 h	24.76 ± 8	28.5	53.9 ± 15	59.9
1323 K for 5.5 h	38.02 ± 9	42.4	79.3 ± 23	87.1

## Data Availability

The authors confirm that the data supporting the findings of this study is available within the article.
